# Adaptive Evolution of Toll-Like Receptors (TLRs) in the Family Suidae

**DOI:** 10.1371/journal.pone.0124069

**Published:** 2015-04-20

**Authors:** Kwame A. Darfour-Oduro, Hendrik-Jan Megens, Alfred L. Roca, Martien A. M. Groenen, Lawrence B. Schook

**Affiliations:** 1 Department of Animal Sciences, University of Illinois, Urbana-Champaign, Illinois, United States of America; 2 Animal Breeding and Genomics Centre, Wageningen University, Wageningen, The Netherlands; Virginia Tech University, UNITED STATES

## Abstract

Members of the family Suidae have diverged over extended evolutionary periods in diverse environments, suggesting that adaptation in response to endemic infectious agents may have occurred. Toll-like receptors (TLRs) comprise a multigene family that acts as the first line of defense against infectious microbes at the host-environment interface. We hypothesized that across the Suidae, positive selection mediated by infectious agents has contributed to the evolution of TLR diversity. Thus, we analyzed *Sus scrofa*, *Sus barbatus*, *Sus verrucosus*, *Sus celebensis*, *Sus scebifrons*, *Babyrousa babyrussa*, *Potamochoerus larvatus*, *Potamochoerus porcus* and *Phacochoerus africanus* genomes. Specifically, analyses were performed to identify evidence of positive selection using Maximum likelihood (ML) methods within a phylogenetic framework for bacterial and viral sensing Suidae TLR extracellular domains. Our analyses did not reveal evidence of positive selection for *TLR3* and *TLR7*, suggesting strong functional conservation among these two genes for members of the Suidae. Positive selection was inferred for Suidae *TLR1*, *TLR2*, *TLR6* and *TLR8* evolution. ML methods identified amino acid sites of the bacterial sensing *TLR1*, *TLR2*, *TLR6* and the viral sensing *TLR8* to be under persistent positive selection. Some of these sites are in close proximity to functionally relevant sites, further strengthening the case for pathogen mediated selection for these sites. The branch leading to the genus *Sus* demonstrated evidence of episodic positive selection for *TLR1*, indicating selection mediated by infectious agents encountered within the specific geographic origin of the *Sus*. These results indicate that species of the Suidae have positively selected residues within functional domains of TLRs reflective of prior infections. Thus, TLR genes represent candidates for experimental validation to determine their functional role in antibacterial and antiviral activity within members of the Suidae.

## Introduction

Bacterial and viral infectious diseases constitute a significant threat to host survival. Host species have developed various strategies to combat these threats, including the development of innate and acquired immune defenses. The innate immune system provides an immediate defensive response against pathogenic infections while the acquired immune system response to pathogenic infections may require weeks to develop. As pathogens evolve to subvert the host immune response, host immune genes evolve in response. The arms race between hosts and microbial pathogens (host-parasite co-evolution) influences variation in the response to infectious disease agents at individuals, population, species levels and within higher order taxonomic units [1].

Adaptive evolution (positive selective) is selective pressure through a change in environment placed on a protein in order to improve the fitness of the organism in that environment [2]. With respect to vertebrate immune-related genes, studies on adaptive evolution have mainly focused on the major histocompatibility complex (MHC), cell surface glycoproteins of the acquired immune system that mediate presentation of peptides to T-cell receptors [3]. In humans, it has been shown that half of the genetic variability in immune response to infections is accounted for by non-MHC genes [4]. Most of these non-MHC genes seem to belong to the innate immune system [5], indicating that such genes may be under adaptive evolution. Phagocytic cells such as monocytes, macrophages and dendritic cells mediate the recognition of pathogens by the innate immune system through germline encoded receptors known as pattern recognition receptors (PRRs). These PRRs recognize conserved molecular features of microbes called pathogen-associated molecular patterns (PAMPs) [6,7]. Among the numerous PRRs, the Toll-like receptor family is the most widely studied. The Toll-like receptors (TLRs) are innate immunity receptors important during early phase of infections and also serve as a link between the innate and acquired immunity during host immune response [8]. Cell surface expressed TLRs (*TLR1*, *TLR2*, *TLR4*, *TLR5* and *TLR6*) recognize predominantly bacterial ligands and several fungal and parasite ligands while *TLR3*, *TLR7* and *TLR8* are expressed within the endosome and recognize single and double-stranded viral RNA [9]. TLRs are type I transmembrane glycoproteins composed of an extracellular domain characterized by a leucine-rich repeat (LRR) motif responsible for binding infectious agents ligands, a transmembrane domain and an intracellular signaling domain.

Previous studies have documented purifying selection [10] and overdominant balancing selection [11] as the dominant selective pressures acting within innate immune genes including TLRs. TLRs might be under positive selection due to a co-evolutionary arms race with their microbial pathogens as they lie directly at the host-environment interface and target microbial molecules [12]. Studies at the interspecies level have found clear signatures of positive selection at codon positions across TLR genes from primate, avian and murinae species [12–15]. In the context of positive selection at the interspecies level, a distinction can be made between persistent positive selection, where selective pressure at codon positions within a gene remains constant throughout time across species and episodic positive selection where selective pressures act in a lineage specific manner [16]. In the case of persistent positive selection, the selective pressure affects most lineages within a phylogeny and is evident as codons rapidly evolving across the species in a phylogenetic tree. For episodic positive selection, codon positions under positive selective pressure within particular lineages may be neutrally or negatively evolving in other lineages. Regardless of the type of selective pressure (persistent or episodic positive selection), detection of evidence of selection of a gene region suggests a selective advantage in changing amino acid sequence in this region [2].

Members of the family Suidae have a widespread distribution. The natural occurrence of *Sus scrofa* (wild boar) is across most of Eurasia while all other species of the genus *Sus* are restricted to Southeast Asia [17]. The *Babyrussa babyrussa* (babyrussa) is also found in Southeast Asia and *Potamochoerus larvatus* (bush pig), *Potamochoerus porcus* (red river hog) and *Phacochoerus africanus* (common warthog) are restricted to sub-Saharan Africa [18]. Such diverse environments of members of the family Suidae suggests adaptation to endemic infectious disease agents may have occurred, that can be investigated as positive selection within TLR genes. However, information on how positive selection has influenced TLR genes within members of the Suidae is limited.

The aim of this study was to determine whether there is evidence of positive selective pressure in the family Suidae in a phylogenetic framework. We hypothesized that positive selection has contributed to the evolution of bacterial and viral sensing TLRs in the family Suidae. The specific aims of this study were to 1) identify evidence of persistent positive selection at TLRs across members of the family Suidae and 2) to determine whether restricted lineages within the Suidae demonstrate TLR positive selection. We focused on the bacterial sensing *TLR1*, *TLR2* and *TLR6* and viral sensing *TLR3*, *TLR7* and *TLR8* as viruses and bacteria are the dominant parasites threatening wild mammals [19]. Identifying positively selected residues within the TLR genes of members of the Suidae will yield vital information as to their adaptation to previous bacterial and viral infections. Our findings suggest that positive selection of TLRs amongst members of the Suidae has been mediated by infectious disease agents.

## Materials and Methods

### Study animals

**Fig 1:**
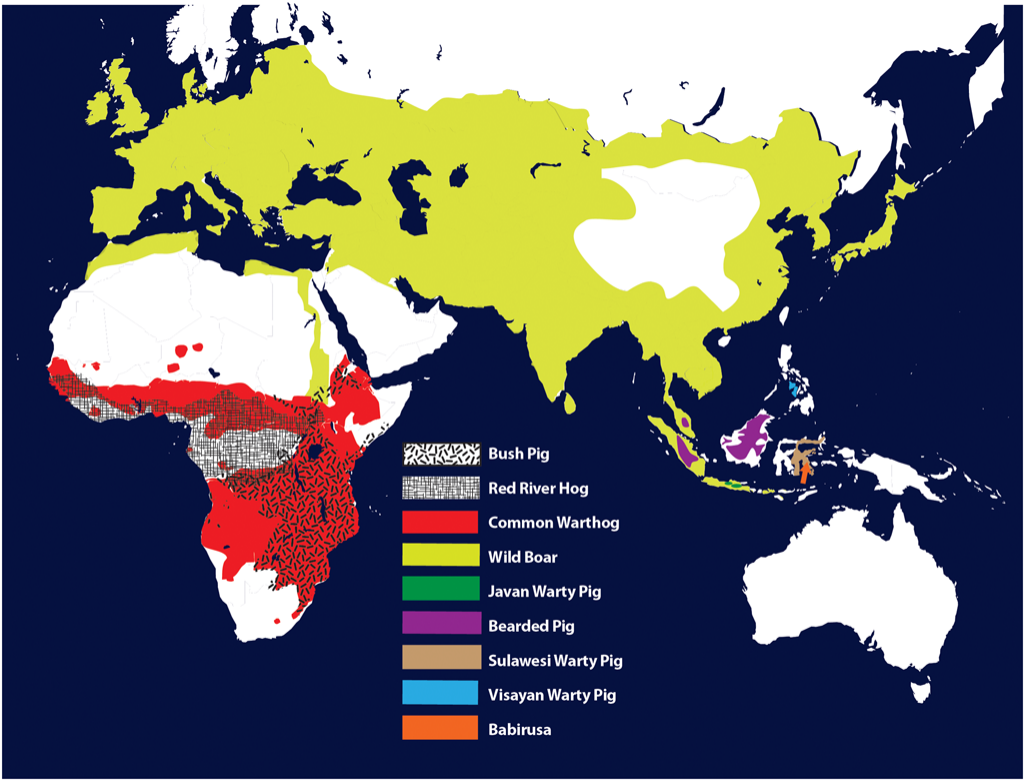
Range map for members of the Suidae.

Ten animals representing 9 species of the family Suidae were utilized in this study. A range map showing the natural distribution of these species is shown in Fig 1. The species *Sus scrofa* (wild boar) was represented by a European wild boar (*Sus scrofa* Europe) and a Asian wild boar (*Sus scrofa* Asia) to reflect the wide distribution of this species. Southeast Asian suids were represented by *Sus verrucosus* (javan warty pig), *Sus celebensis* (sulawesi warty pig), *Sus scebifrons* (visayan warty pig), *Sus barbatus* (bearded pig) and *Babyrousa babyrussa* (babirusa). Suidae species of African origin were represented by *Potamochoerus larvatus* (bush pig), *Potamochoerus porcus* (red river hog) and *Phacochoerus africanus* (common warthog).

### Genes analyzed

*TLR1*, *TLR2* and *TLR6* encoding receptors for bacterial ligands and *TLR3*, *TLR7* and *TLR8* recognizing viral ligands were selected for this study. The extracellular domains were the focus since they encode the functional sites involved in pathogen ligand recognition.

### DNA extraction and sequencing

DNA extraction, library preparation and sequencing was performed as previously described [20]. Briefly, DNA was extracted from whole blood by using the QIAamp DNA blood spin kit (Qiagen Sciences) and quantity and quality parameters were performed on the Qubit 2.0 fluorometer (Invitrogen) and run on a 1% agarose gel. Library construction and re-sequencing of individual members of the family Suidae were done with 1–3 ug of genomic DNA according to the Illumina library prepping protocols. The library insert size was 300–500 bp and sequencing was performed using a 100 paired-end sequencing kit [20]. All DNA were sequenced to approximately 8x depth. Quality trimmed reads (phred quality>20, minimum length of pairs of reads = 40bp) were aligned to the *Sus scrofa* reference genome build 10.2 [21] using the unique alignment option of Mosaik Aligner (V.1.1.0017). The aligned reads from each of the animals together with the *Sus scrofa* reference genome were stored as bam files for each individual animal.

### Orthologs identification and delineation of their extracellular domains

Porcine TLR mRNA sequences were obtained from Ensemble database (http://www.ensemble.org). The accession numbers of sequences obtained from the public databases were *TLR1*: NM_001031775, *TLR2*: NM_213761, *TLR3*: HQ412796, *TLR6*: NM_213760, *TLR7*: NM_001097434, *TLR8*: ENSSSCG00000012118. When a TLR gene was found to have more than one transcript, the longest transcript was chosen. The genomic coordinates of the porcine TLR mRNA sequences within the *Sus scrofa* genome assembly 10.2 were obtained from Ensemble. Based on these genomic coordinates, sequences of TLR gene orthologs were then retrieved from aligned bam files (illumina resequencing data for family Suidae species aligned against *Sus scrofa* genome assembly 10.2) of *Sus scrofa* (*Sus scrofa* Europe and *Sus scrofa* Asia), *Sus verrucosus*, *Sus celebensis*, *Sus scebifrons*, *Sus barbatus Babyrousa babyrussa*, *Potamochoerus larvatus*, *Potamochoerus porcus* and *Phacochoerus africanus* to identify TLR gene orthologs. The resulting sequences for each species were then blast screened against the *Sus scrofa* genome to ensure similarity with the porcine TLR mRNA sequences. Exonic regions were then obtained from these sequences and concatenated to obtain coding sequences. The coding sequences were further trimmed to obtain sequences of the extracellular domain for each TLR in each species. Sequences were aligned using ClustalW 1.81 [22]. In this study, porcine TLR reference amino acid sequences were aligned to corresponding human and murine sequences in order to delineate the extracellular domains of porcine TLRs and their LRR modules and sub-domains [23–28] (S1 Fig). The genomic coordinates of the TLR extracellular domains are provided in S1 Table.

### ML test for positive selection

Comparison of the non-synonymous substitutions per non-synonymous site (*dN*) with the number of synonymous substitutions per synonymous site (*dS*) in a maximum likelihood (ML) framework was used to test for positive selection for every codon, defining a *dN*/*dS* ratio (ω)>1 in a codon as evidence of positive selection. First, we determined whether ω varied among codon sites for each TLR alignment by comparing CODEML program models in PAML version 4 [29,30] M0 which assumes that ω is constant across all sites in the alignment and M3 which allows ω to vary amongst sites.

Next, 4 site models were employed to detect sites under persistent positive selection. Two models each from the CODEML program in PAML version 4 [29,30] and the Datamonkey web server [31] were utilized. CODEML site model M1a, a nearly neutral evolution model where sites are assumed to be evolving under either purifying selection (ω<1) or neutral evolution (ω = 1) was compared to model M2a that allows positive selection among sites. M7, which allows sites to evolve under either purifying selection or neutrally, was compared to model M8, which allows for positively selected sites. Models M7 and M8 differ from models M1a and M2a in that, the former assume that ω values are drawn from a beta distribution [32]. Models were compared using a likelihood ratio test (LRT). In order to identify positive selection, twice the difference in log-likelihood values (2lnΔL) between models would be significant by chi-square testing. The F3x4 model of codon frequencies was used for the analyses. Models were run in duplicates with ω of 0.5 and 1.5 to increase the probability of convergence of model parameters. The Bayes empirical Bayes (BEB) approach implemented in CODEML was used to identify codons under positive selection. BEB estimates the posterior probability of each site belonging to three selection classes: low, intermediate and high ω. Codon sites with ω>1 and a posterior probability >95% were inferred to be under positive selection. Fixed-effects likelihood (FEL) and Random-effects likelihood (REL) models implemented using the Datamonkey web server were also used to detect positive selection. The FEL model estimates synonymous and nonsynonymous rates directly at each codon site, without assuming an a priori distribution of rates across sites while REL model allows synonymous and nonsynonymous substitution rates to vary among codon sites. Codon sites were considered to be under positive selection at significant levels p<0.1 for FEL and a Bayes factor >50 for REL [33].

**Fig 2:**
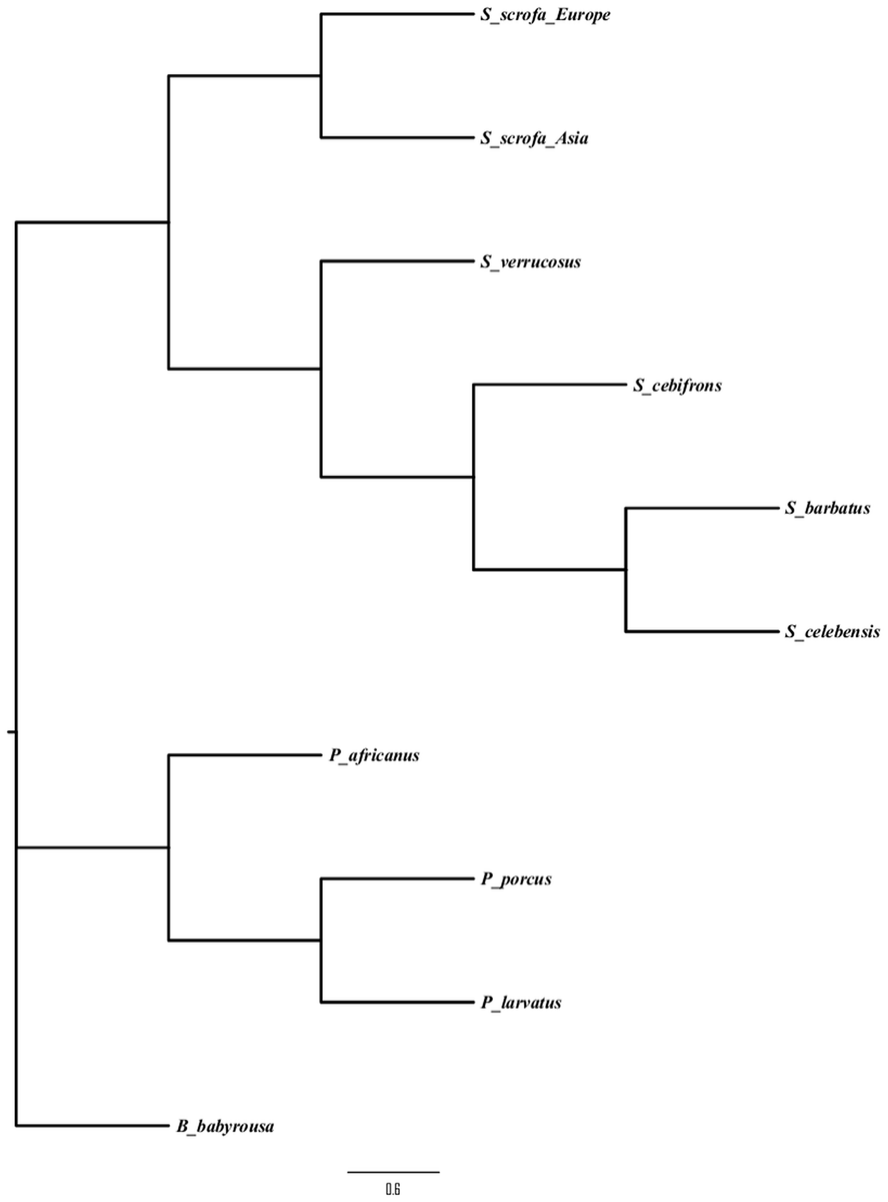
Species phylogeny of the Suidae. Shown here is a representation of the relationships among members of the Suidae used in analyses. The relationships were derived from near complete genome data of each species. The posterior probability at each node is 1.

To test for episodic positive selection, Branch-Site REL and MEME (mixed effects model of evolution) implemented on the Datamonkey web server were utilized. The Branch-Site REL model estimates proportion of sites under selection along tree branches and allows evolutionary rates to simultaneously vary along phylogenetic branches and sites [16]. The MEME method identifies lineage-specific events of positive selection at sites, even though the same site is under purifying or neutral selection in other lineages [34]. A Suidae species tree (Fig 2) derived from near complete genome sequences for members of the Suidae [17] [L. Frantz, personal communication, September 27, 2014] was used in all analyses.

Positively selected sites detected in this study were compared to human TLR Swiss-Prot database to determine their possible link to function. Sites under positive selection were also mapped to three dimensional (3D) protein structures using MuPIT Interactive [35] in order to examine their functional significance. We also determined the conservative or radical nature of amino acid changes at sites under positive selection within this study.

## Results

The sequences (10 sequences within each TLR alignment) of the extracellular domains of bacterial sensing *TLR1*, *TLR2* and *TLR6* and viral sensing *TLR3*, *TLR7* and *TLR8* from species within the family Suidae were obtained. The length of the extracellular domains in terms of number of nucleotides of the TLRs ranged from 1668 bases for *TLR1* to 2445 bases for *TLR7*. Amino acid length ranged from 556 amino acids for *TLR1* to 792 amino acids for *TLR7*.

### Heterogeneity of selective pressure along genes

To determine whether selective pressures varied amongst codon sites for each TLR gene, the M0 and M3 models of CODEML program was utilized. Comparison of M0 vs M3 indicated that *dN*/*dS* ratio (ω) of some TLR genes varied among codons, implying that selective constraints were heterogeneous between sites. We detected significant (p<0.01 for 2lnΔl) heterogeneity of ω along *TLR1*, *TLR2*, *TLR6* and *TLR8* (Table 1). For these genes, we found that the proportion of sites with evidence of positive selection (p_2_) is relatively smaller than the proportion of sites with evidence of purifying (p_0_) or neutral (p_1_) selection. Thus, the majority of sites within the proteins of *TLR1*, *TLR2*, *TLR6* and *TLR8* were functionally constrained. *TLR3* and *TLR7* sequences did not reveal heterogeneity of selection pressure ω among their codons and are thus functionally conserved along their entire extracellular domains within the members of Suidae involved in this study.

**Table 1:** Test of heterogeneity of selective pressure along genes.

Gene	Model	Parameter estimates	Log likelihood (L)	2lnΔL^a^
*TLR1*	M0	ω = 0.3788	-2648.2197	35.9222**
	M3	p_0_ = 0.2608, ω_0_ = 0.2002, p_1_ = 0.7235, ω_1_ = 0.2002, p_2_ = 0.0157, ω_2_ = 20.3741	-2630.2586	
*TLR2*	M0	ω = 0.4383	-2762.6134	17.2620**
	M3	p_0_ = 0.0695, ω_0_ = 0.2757, p_1_ = 0.9142, ω_1_ = 0.2757, p_2_ = 0.0163, ω_2_ = 11.1174	-2753.9824	
*TLR3*	M0	ω = 0.2649	-2952.9019	0.0000^NS^
	M3	p_0_ = 0.2240, ω_0_ = 0.2649, p_1_ = 0.3124, ω_1_ = 0.2650, p_2_ = 0.4635, ω_2_ = 0.2650	-2952.9019	
*TLR6*	M0	ω = 0.4692	-2695.5056	30.8540**
	M3	p_0_ = 0.3864, ω_0_ = 0.0000, p_1_ = 0.5357, ω_1_ = 0.0000, p_2_ = 0.0780, ω_2_ = 6.7044	-2680.0786	
*TLR7*	M0	ω = 0.1087	-3980.3407	1.2288^NS^
	M3	p_0_ = 0.4460, ω_0_ = 0.0000, p_1_ = 0.2772, ω_1_ = 0.0000, p_2_ = 0.2768, ω_2_ = 0.3977	-3979.7263	
*TLR8*	M0	ω = 0.3085	-3985.5001	21.3048**
	M3	p_0_ = 0.7187, ω_0_ = 0.0000, p_1_ = 0.1417, ω_1_ = 0.0000, p_2_ = 0.1397, ω_2_ = 2.3685	-3974.8477	

p_0_ is proportion of sites where ω <1(ω_0_), p_1_ is proportion of sites where ω = 1(ω_1_).

p_2_ is proportion of sites where ω>1(ω_2_).

^a^ Twice the difference in log-likelihood values between models M0 and M3.

**p<0.01.

^NS^Not significant.

### Detection of persistent positive selection across members of the Suidae

To detect positive selection pressure that have acted persistently and shared across most Suidae members regardless of their geographic origins, site models implemented in the CODEML program of the PAML package and on the Datamonkey web server were utilized. Site models permit detection of positive selection within gene codons. Site models detected positively selected codons in bacterial sensing *TLR1*, *TLR2* and *TLR6* and viral sensing *TLR8*. Specifically, comparisons of nested models available in CODEML program indicated that models including codons with ω>1 (M2a and M8) demonstrated a better fit than did neutral models (M1a and M7) for all the four TLR genes (S2 and S3 Tables). Since detecting codons under positive selection using site-based methods have power limitations when analyzing a few closely related species [36], we defined sites under positive selection conservatively as those for which significant results were obtained by more than one site model. Such sites and the properties of their amino acids are shown in Table 2. Three codons were identified for *TLR1*, 2 codons for *TLR2*, 7 codons for *TLR6* and 2 codons for *TLR8* that showed evidence for persistent positive selection. The site based methods did not identify codons under positive selective pressure for *TLR3* and *TLR7*.

**Table 2:** Positively selected codons within the extracellular domains of TLRs.

Species	Origin	Genes														
		*TLR1*				*TLR2*		*TLR6*							*TLR8*	
		Codons														
		117	434*	451	559	216	338*	183	334	452	459	501	554	560	178	388
*S*. *scrofa* (E)	Eurasia	Met	Leu	Ile	Glu	Lys	Ala	Met	Arg	Ile	Ser	Thr	Ser	Glu	Asp	Phe
*S*. *scrofa* (A)		Met	Leu	Ile	Glu	Lys	Ala	Met	Arg	Ile	Asn	Ile	Gly	Lys	Glu	Phe
*S*. *barbatus*	Southeast Asia	Thr	Leu	Val	Glu	Lys	Thr	X	Arg	Ile	Asn	X	Gly	Glu	Asp	Phe
*S*. *celebensis*		Thr	Leu	Val	Lys	Glu	Thr	Met	Gly	Val	X	Ile	Gly	Glu	Glu	Phe
*S*. *cebifrons*		Thr	Leu	X	X	Lys	Thr	Thr	Arg	Val	Asn	Thr	Gly	Glu	Glu	Phe
*S*. *verrucosus*		Thr	Met	Ile	X	Glu	Thr	Met	Gly	Ile	X	Ile	Ser	Glu	Glu	Phe
*B*. *babyrussa*		Ile	Met	Val	Lys	Glu	Ala	Val	Arg	Val	Ser	Ile	Gly	Lys	Glu	Phe
*P*. *larvatus*	Africa	Thr	Met	Val	Glu	Glu	Ala	Val	Arg	Val	Asn	Ile	Gly	Glu	Glu	Leu
*P*. *porcus*		Thr	Met	Val	Glu	Glu	Lys	Val	Arg	Val	Asn	Ile	Gly	Glu	Glu	Phe
*P*. *africanus*		Thr	Met	Val	Lys	Glu	Ala	Val	Arg	Val	Asn	Ile	Gly	Glu	Glu	Val

*Codon site 434 in TLR1 is under episodic positive selection and codon site 338 in TLR2 is under both persistent and episodic positive selection. Other codon sites are under persistent positive selection. Amino acid properties: Met, Ile, Leu, Val, Ala are non-polar aliphatic; Thr, Ser, Asn are polar neutral; Lys, Arg are polar positive; Glu, Asp are polar negative; Gly is non-polar neutral; Phe is non-polar aromatic. (E) represents Europe, (A) represents Asia. X represents an undetermined amino acid.

### Detection of episodic positive selection in particular lineages

To detect signatures of episodic positive selection in specific lineages for each TLR gene, branch-site REL analysis available on the Datamonkey web server were performed. The branch-site REL identify lineages at which a proportion of sites have *dN*/*dS* ratios >1without making any assumptions as to which lineages should be analyzed for positive selection. With respect to *TLR1*, evidence for positive selection in the ancestral lineage of the genus *Sus* (internal branch leading to the *Sus* clade) and on *TLR2* species branch corresponding to *Sus verrucosus* (Table 3) were detected. Analyses also indicated that within the TLR2 gene, the species branch corresponding to *Potamochoerus porcus* is under positive selective pressure (Table 3). Thus, MEME was employed to identify sites under positive selection along branches. One codon position in *TLR1* (codon position 434) in the lineage leading to the genus *Sus* and the species branch corresponding to *Sus verrucosus* was identified as under positive selection. Another codon position (codon position 338 in *TLR2*) was found to be under positive selection in the species branch corresponding to *Potamochoerus porcus* (Table 3).

**Table 3:** Branches and codons under lineage specific positive selection in family Suidae.

Branch-site REL analysis					MEME analysis	
Gene	Branch	ω^+^	Pr[ω = ω^+^]	p value	Codons	p value
*TLR1*	Ancestral lineage of *Sus*	1012.60	0.0002	0.003	434	0.090
	*S*.*verrucosus*	785.40	0.0002	0.003	434	0.090
*TLR2*	*P*. *porcus*	3334.61	0.0001	0.001	338	0.002

ω+ represents the ω value inferred for positively selected sites along branch; Pr[ω = ω+]represents the proportion of sites inferred to be evolving at ω+ along branch; p represents the p-value for episodic selection at branch corrected for multiple testing using the Holm-Bonferroni method.

### Functional significance of positively selected sites

**Fig 3:**
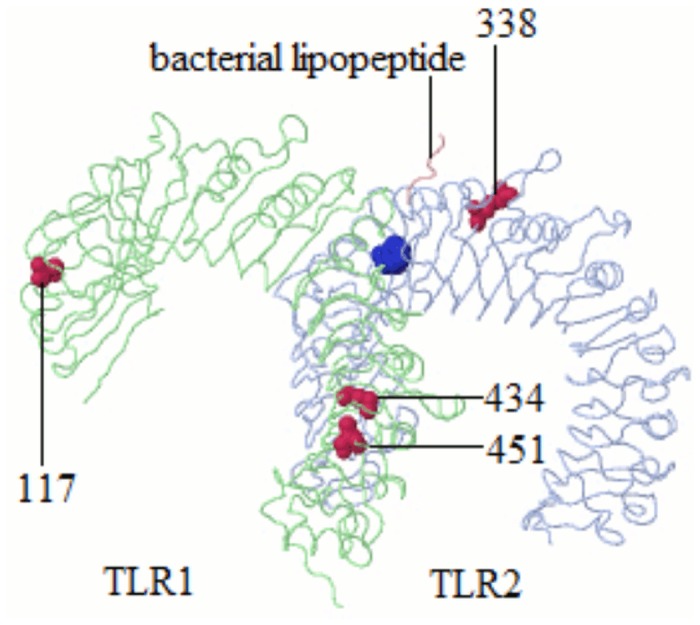
Positively selected sites in the three dimensional structure of TLR1/TLR2 heterodimer. Positively selected sites are colored in red. A site known to interact with bacterial lipopeptide is colored in blue. Only sites likely to affection protein function based on their location within the structure are shown.

To determine functional relevance of positively selected amino acid sites, sites determined to be under positive selection by more than one ML method were compared to human TLR Swiss-Prot entries (Table 4). First, human and porcine TLRs were aligned to determine the equivalent positions of positively selected sites in pigs within humans. Then analysis were performed to determine whether the sites in human TLRs have been implicated as having functional effects or are in close proximity to a functionally annotated site from human Swiss-Prot. Sites that were adjacent to residues and within regions known to affect TLR protein function (Table 4) were detected. Thus, amino acid sites under positive selection, as determined by more than one ML method were mapped onto TLR protein 3D crystallographic structures to gain further insight into their functional significance. Positively selected sites were within the following domains: *TLR1* 117 (LRR4), 434 (LRR16), 451 (LRR17), 559 (LRR carboxy termini (LRRCT)); *TLR2* 216 (LRR7), 338 (LRR12); *TLR6* 183 (LRR6), 334 (LRR12), 452 and 459 (LRR17), 501(LRR19), 554 and 560 (LRRCT); *TLR8* 178 (LRR5), 388 (LRR13). Positively selected sites which can be inferred to affect protein function based on their location within TLR protein 3D crystallographic structures are shown in Fig 3. Two of the positively selected codons (*TLR1* sites 434 and 451) are within *TLR1*/*TLR2* interface (Fig 3) and might have implications for *TLR1*/*TLR2* heterodimer formation. *TLR2* site 338 is in close proximity to a site that interacts with bacterial lipopeptides and may therefore have a role in ligand binding. The conservative or radical nature of amino acid changes occurring at positively selected sites was also determined. Radical amino acid changes have effects on protein function. Specific sites (*TLR1*:117, 559; *TLR2*:216, 338; *TLR6*:183, 334, 501, 554, 560; *TLR8*:388) have experienced radical amino acid changes (Table 2), suggesting a possible role of such sites in diverse protein functions.

**Table 4:** Positively selected sites predicted to affect TLR function based on human Swiss Prot entries and site location in three dimensional TLR structures.

Gene	Codon	Equivalent codon in human	Functional Information
*TLR1*	117	113	In the region (110–132) of cysteine residues participating in disulphide bonds^a^
	434	430	Adjacent to a glycosylation site (429)^a^
	559	555	Adjacent to site of SNP (Tyr554Cys) that leads to marked impairment of NF-kB activation^b^
*TLR2*	338	337	LRR12—involved in ligand binding^a^
*TLR6*	334	334	LRR12—involved in TLR2-TLR6 heterodimerization^c^

^a, b, c^ represent references:

^a^ [22],

^b^[41],

^c^ [24].

## Discussion

The important role of pathogen mediated positive selection pressure in shaping diversity in the TLRs of mammalian species has been documented elsewhere [12,13,37]. The adaptation of the members of the Suidae to different environments presenting numerous bacterial and viral pathogenic challenges, make the family amenable to studies of pathogen mediated selection on immune genes. Results obtained in this study indicate that both persistent and episodic positive selection have shaped TLR evolution and diversity among the Suidae.

Our finding of small proportion of sites of *TLR1*, *TLR2*, *TLR6* and *TLR8* showing evidence of persistent positive selection agrees with the mostly accepted paradigm that purifying selection is the dominant force operating on TLRs [10,12]. As was the case with previous studies [12,15,38], more positively selected sites within bacterial-sensing TLRs than their viral-sensing counterparts were inferred. Viral infections are thought to exert stronger selective pressure than bacterial infections, constraining the evolution of viral-sensing TLRs [38]. In contrast to previous studies done in primates [12] and across rodents, carnivores, lagomorphs and primates [37], fewer sites under persistent positive selection within genes involved in this study were detected. Members of the Suidae represent closely related species and are therefore likely to be affected by fewer related bacteria and viruses than the diverse species involved in previous studies. *TLR6* stood out as the gene with the strongest evidence of selection, where more codons were under persistent positive selection. The dimerization interface in *TLR6*/*TLR2* is 80% larger than that of *TLR1*/*TLR2* [39]. Therefore one can speculate that the larger *TLR6*/*TLR2* dimerization surface exposes more codons of *TLR6* to positive selective pressure. The finding that among the bacterial sensing TLRs, *TLR2* had fewer sites under persistent positive selection despite having similar protein length as *TLR1* and *TLR6* is suggestive of a stronger selective constraint on *TLR2*. The *TLR2* gene product recognizes a myriad of ligands (microbial triacyl lipoproteins, diacyl lipoproteins found in mycoplasma, lipoteichoic acid of Gram-positive bacteria or Zymosan of yeast) pathogens through heterodimerization with *TLR1* and *TLR6* [40,41]. *TLR2* also been shown to affect IFN production, making the *TLR2* gene evolutionary constrained [42].

Apart from persistent pathogen mediated positive selection acting over long evolutionary time across members of the Suidae, the evolutionary histories of members of the Suidae may have been affected by periodic pathogenic infections confined to specific lineages within certain geographic locations, leading to episodic positive selection within such lineages. Such a signal of adaptive evolution is usually masked by a background signal of purifying selection, which makes their identification difficult. Both Branch Site REL and the MEME methodology implemented in Datamonkey revealed the same lineages were evolving under episodic positive selection, suggesting that sites within these branches are under positive selection. MEME is a recently developed method [34] that allows the detection of episodic positive selection even when majority of lineages are evolving under purifying selection.

Our lineage specific analysis showed that the branch leading to the *Sus* clade was found to have undergone episodic positive selection at *TLR1* amino acid site 434 indicating that the ancestors of species within the genus *Sus* had to undergo adaptive changes at this site in response to their environment. With the exception of *Sus verrucosus* which had methionine at *TLR1* site 434, other *Sus* species had the leucine residue while the African suids and *Babyrousa babyrussa* had methionine. This finding suggests a possible selective advantage for leucine at *TLR1* site 434 in the environment in which ancestors of the *Sus* species originated. Indeed, methionine seems to be very rare at *TLR1* site 434 within the domesticated breeds of *Sus scrofa* [43], indicating leucine is preferred at this site. The substitutions of methionine with leucine within the interior of a protein increase protein stability [44] supporting a hypothesis that leucine within the *Sus* species stabilizes the *TLR1* protein prior to heterodimerization with *TLR2* for efficient recognition of diverse bacterial ligands (peptidoglycans and triacyl lipoproteins). This finding of positive selection on branches leading to *Sus verrucosus* for *TLR1* and *Potamochoerus porcus* for *TLR2* requires a cautious interpretation, since only one sequence from one animal is involved in each case. *Sus verrucosus* is thought to represent a distinct lineage following a deep split with other species of the genus *Sus* [17]. It is possible that bacterial pathogens restricted to *Sus verrucosus* may have exerted selective pressure on its *TLR1* gene. Related to *Potamochoerus porcus*, positive selection on *TLR2* gene could partly be due to adaptation to infectious agents within the African rain forest, a location outside of which they are rarely found [45].

The case for positive selection within TLR amino acid sites involved in this study is strengthened by the location of specific sites in close proximity to functionally relevant regions. Site 117 of *TLR1* is within disulphide bonds region. Disulphide bonds are important to the overall function of proteins as they are associated with their folding and stability [46]. Site 434 of *TLR1* is adjacent to a glycosylation site. One conclusion would be that positive selection at this site is of consequence as glycosylation of TLRs is thought to influence receptor surface presentation, trafficking and ligand recognition [47]. The positive selection inferred at site 559 of *TLR1*, adjacent to a site that leads to impairment of NF-kB activation, suggests a role for this site in regulating inflammatory response to bacterial infection.

*TLR1*/*TLR2* heterodimer formation is required for ligand recognition and signal initiation [23]. Thus changes at sites 434 and 559 within *TLR1* suggest residues at these sites could be under selective pressure to improve the *TLR1/TLR2* heterodimer formation…. As was the case with the study of [48] involving RIG-I-like pattern recognition receptors, in this study the majority (10/15 sites) of sites under positive selection involved radical amino acid residue changes across species of the Suidae. This is in agreement with positive selection favoring radical amino changes at sites within particular genes [49]. Such sites may be of functional significance.

Results obtained here have implications for present day domestic pigs. African wild suids are susceptible to some viral, bacterial and parasitic diseases of domestic pigs. As European and Asian wild boars are the progenitors of most domestic pigs, it is likely that species of the genus *Sus* are also susceptible to diseases of domestic swine [50]. Thus, residues that were under positive selection in the past could still be beneficial to domestic pigs in terms of disease resistance. Evidence for past positive selection influencing resistance or susceptibility to present day pathogens is seen in the Protein Kinase R (PKR) gene, where adaptive changes at important residues, most likely driven by old viruses [51], are important in the ability of PKR to fight infections from present-day poxyviruses [52].

## Conclusion

In conclusion, residues within bacterial sensing *TLR1*, *TL2*, *TLR6* and viral sensing *TLR8* of members of the Suidae that have undergone persistent and episodic positive selection were identified. The evidence of positive selection on the TLR genes reveals that pathogen mediated selective pressure has shaped Suidae TLR evolution. The case for positive selective at amino acid sites is strengthened by location of these sites in close proximity to functionally relevant sites and the radical changes in amino acids at some of these sites across members of the Suidae. Sites under positive selection may have aided in the adaptation of the Suidae to infectious agents that evolved rapidly or that were encountered in new environments.

## Supporting Information

S1 Fig Domain characterization of TLRs. / ligand binding residues, d residues involved in dimerization, + residues involved in both ligand binding and dimerization. LRR represents leucine rich repeat. LRRNT represents LRR amino termini. LRRCT represents LRR carboxy termini. Human TLR sequences accession numbers: *TLR1*:Q15399; *TLR2*:O60603; *TLR3*: NP_003256.1; *TLR6*:Q9Y2C9; *TLR7*:NP_057646; *TLR8*:NP_619542. Porcine TLR sequences accession numbers: *TLR1*:NP_001026945; *TLR2*:NP_998926.1; *TLR3*:DQ266435.1 *TLR6*:NP_998925.1; *TLR7*: DQ647699; *TLR8*: NP_999352.1. For *TLR6*, human LRRs were determined from their alignment with murine *TLR6*. Asterisks, colons and periods under aligned the aligned sequences indicate complete match, strong conservation and weaker conservation of amino acid respectively.
(DOCX)

S1 File Compressed zip file containing sequence alignment in fasta format used in this study. (ZIP)

S1 Table Summary of TLR extracellular domains studied. ^a^Exon encoding the extracellular domain.
(DOCX)

S2 Table Results from site models at codons under persistent positive selection in TLRs. (DOCX)

S3 Table Parameter estimates for PAML models used in detecting persistent positive selection in TLRs. (DOCX)

## References

[1] Dawson HD, Loveland JE, Pascal G, Gilbert JGR, Uenishi H, Mann KM, et al Structural and functional annotation of the porcine immunome. BMC Genomics. 2013;14: 332 Available: http://www.pubmedcentral.nih.gov/articlerender.fcgi?artid=3658956&tool=pmcentrez&rendertype=abstract. 10.1186/1471-2164-14-332 23676093 PMC3658956

[2] Morgan CC, Loughran NB, Walsh TA, Harrison AJ, O’Connell MJ. Positive selection neighboring functionally essential sites and disease-implicated regions of mammalian reproductive proteins. BMC Evol Biol. 2010;10: 39 Available: http://www.biomedcentral.com/1471-2148/10/39. 10.1186/1471-2148-10-39 20149245 PMC2830953

[3] Klein J, Sato A. The HLA system. First of two parts. N Engl J Med. 2000;343: 702–9. Available: http://www.ncbi.nlm.nih.gov/pubmed/10974135. 10974135 10.1056/NEJM200009073431006

[4] Jepson A, Banya W, Sisay-Joof F, Hassan-King M, Nunes C, Bennett S, et al Quantification of the relative contribution of major histocompatibility complex (MHC) and non-MHC genes to human immune responses to foreign antigens. Infect Immun. 1997;65: 872–6. Available: http://www.pubmedcentral.nih.gov/articlerender.fcgi?artid=175062&tool=pmcentrez&rendertype=abstract. 9038290 10.1128/iai.65.3.872-876.1997 PMC175062

[5] Fornuskova A, Bryja J, Vinkler M, Macholán M, Piálek J. Contrasting patterns of polymorphism and selection in bacterial-sensing toll-like receptor 4 in two house mouse subspecies. Ecol Evol. 2014;4: 2931–44. Available: http://www.pubmedcentral.nih.gov/articlerender.fcgi?artid=4130449&tool=pmcentrez&rendertype=abstract. 10.1002/ece3.1137 25165529 PMC4130449

[6] Medzhitov R. Recognition of microorganisms and activation of the immune response. Nature. Nature Publishing Group; 2007;449: 819–26. Available: 10.1038/nature06246. 17943118

[7] Kawai T, Akira S. The role of pattern-recognition receptors in innate immunity: update on Toll-like receptors. Nat Immunol. Nature Publishing Group; 2010;11: 373–84. Available: 10.1038/ni.1863. 10.1038/ni.1863 20404851

[8] Werling D, Jungi TW. TOLL-like receptors linking innate and adaptive immune response. Vet Immunol Immunopathol. 2003;91: 1–12. Available: http://www.ncbi.nlm.nih.gov/pubmed/12507844. 12507844 10.1016/s0165-2427(02)00228-3

[9] Akira S, Uematsu S, Takeuchi O. Pathogen recognition and innate immunity. Cell. 2006;124: 783–801. Available: http://www.ncbi.nlm.nih.gov/pubmed/16497588. 16497588 10.1016/j.cell.2006.02.015

[10] Mukherjee S, Sarkar-Roy N, Wagener DK, Majumder PP. Signatures of natural selection are not uniform across genes of innate immune system, but purifying selection is the dominant signature. Proc Natl Acad Sci U S A. 2009;106: 7073–8. Available: http://www.pubmedcentral.nih.gov/articlerender.fcgi?artid=2678448&tool=pmcentrez&rendertype=abstract. 10.1073/pnas.0811357106 19359493 PMC2678448

[11] Ferrer-Admetlla A, Bosch E, Sikora M, Marquès-Bonet T, Ramírez-Soriano A, Muntasell A, et al Balancing selection is the main force shaping the evolution of innate immunity genes. J Immunol. 2008;181: 1315–22. Available: http://www.ncbi.nlm.nih.gov/pubmed/18606686. 18606686 10.4049/jimmunol.181.2.1315

[12] Wlasiuk G, Nachman MW. Adaptation and constraint at Toll-like receptors in primates. Mol Biol Evol. 2010;27: 2172–86. Available: http://www.pubmedcentral.nih.gov/articlerender.fcgi?artid=3107592&tool=pmcentrez&rendertype=abstract. 10.1093/molbev/msq104 20410160 PMC3107592

[13] Alcaide M, Edwards S V. Molecular evolution of the toll-like receptor multigene family in birds. Mol Biol Evol. 2011;28: 1703–15. Available: http://www.ncbi.nlm.nih.gov/pubmed/21239391. 10.1093/molbev/msq351 21239391

[14] Grueber CE, Wallis GP, Jamieson IG. Episodic positive selection in the evolution of avian toll-like receptor innate immunity genes. Ellegren H, editor. PLoS One. Public Library of Science; 2014;9: e89632 Available: http://dx.plos.org/10.1371/journal.pone.0089632. 10.1371/journal.pone.0089632 24595315 PMC3940441

[15] Fornůsková A, Vinkler M, Pagès M, Galan M, Jousselin E, Cerqueira F, et al Contrasted evolutionary histories of two Toll-like receptors (Tlr4 and Tlr7) in wild rodents (MURINAE). BMC Evol Biol. 2013;13: 194 Available: http://www.biomedcentral.com/1471-2148/13/194. 10.1186/1471-2148-13-194 24028551 PMC3848458

[16] Kosakovsky Pond SL, Murrell B, Fourment M, Frost SDW, Delport W, Scheffler K. A random effects branch-site model for detecting episodic diversifying selection. Mol Biol Evol. 2011;28: 3033–43. Available: http://www.pubmedcentral.nih.gov/articlerender.fcgi?artid=3247808&tool=pmcentrez&rendertype=abstract. 10.1093/molbev/msr125 21670087 PMC3247808

[17] Frantz LAF, Schraiber JG, Madsen O, Megens H-J, Bosse M, Paudel Y, et al Genome sequencing reveals fine scale diversification and reticulation history during speciation in Sus. Genome Biol. 2013;14: R107 Available: http://genomebiology.com/2013/14/9/R107. 24070215 10.1186/gb-2013-14-9-r107 PMC4053821

[18] Grubb P. Order Artiodactyla In: Wilson DE, Reeder DM, editors. Mammal species of the world: a taxanomic and geographic reference. 3rd ed Baltimore, Maryland: Johns Hopkins University Press; 2005 pp. 637–772.

[19] Pedersen A, Jones K, Nunn C, Altizer S. Infectious diseases and extinction risk in wild mammals [Internet]. CONSERVATION BIOLOGY. 2007 Available: http://discovery.ucl.ac.uk/1347949/. 10.1111/j.1523-1739.2007.00776.x PMC7202242 17883492

[20] Bosse M, Megens H-J, Madsen O, Paudel Y, Frantz LAF, Schook LB, et al Regions of homozygosity in the porcine genome: consequence of demography and the recombination landscape. PLoS Genet. 2012;8: e1003100 Available: http://www.pubmedcentral.nih.gov/articlerender.fcgi?artid=3510040&tool=pmcentrez&rendertype=abstract. 10.1371/journal.pgen.1003100 23209444 PMC3510040

[21] Groenen MAM, Archibald AL, Uenishi H, Tuggle CK, Takeuchi Y, Rothschild MF, et al Analyses of pig genomes provide insight into porcine demography and evolution. Nature. 2012;491: 393–8. Available: http://www.pubmedcentral.nih.gov/articlerender.fcgi?artid=3566564&tool=pmcentrez&rendertype=abstract. 10.1038/nature11622 23151582 PMC3566564

[22] Thompson JD, Gibson TJ, Plewniak F, Jeanmougin F, Higgins DG. The CLUSTAL_X windows interface: flexible strategies for multiple sequence alignment aided by quality analysis tools. Nucleic Acids Res. 1997;25: 4876–82. Available: http://www.pubmedcentral.nih.gov/articlerender.fcgi?artid=147148&tool=pmcentrez&rendertype=abstract. 9396791 10.1093/nar/25.24.4876 PMC147148

[23] Jin MS, Kim SE, Heo JY, Lee ME, Kim HM, Paik S-G, et al Crystal structure of the TLR1-TLR2 heterodimer induced by binding of a tri-acylated lipopeptide. Cell. 2007;130: 1071–82. Available: http://www.ncbi.nlm.nih.gov/pubmed/17889651. 17889651 10.1016/j.cell.2007.09.008

[24] Liu L, Botos I, Wang Y, Leonard JN, Shiloach J, Segal DM, et al Structural basis of toll-like receptor 3 signaling with double-stranded RNA. Science. 2008;320: 379–81. Available: http://www.pubmedcentral.nih.gov/articlerender.fcgi?artid=2761030&tool=pmcentrez&rendertype=abstract. 10.1126/science.1155406 18420935 PMC2761030

[25] Kang JY, Nan X, Jin MS, Youn S-J, Ryu YH, Mah S, et al Recognition of lipopeptide patterns by Toll-like receptor 2-Toll-like receptor 6 heterodimer. Immunity. 2009;31: 873–84. Available: http://www.ncbi.nlm.nih.gov/pubmed/19931471. 10.1016/j.immuni.2009.09.018 19931471

[26] Wei T, Gong J, Jamitzky F, Heckl WM, Stark RW, Rössle SC. Homology modeling of human Toll-like receptors TLR7, 8, and 9 ligand-binding domains. Protein Sci. 2009;18: 1684–91. Available: http://www.pubmedcentral.nih.gov/articlerender.fcgi?artid=2776956&tool=pmcentrez&rendertype=abstract. 10.1002/pro.186 19521997 PMC2776956

[27] Tanji H, Ohto U, Shibata T, Miyake K, Shimizu T. Structural reorganization of the Toll-like receptor 8 dimer induced by agonistic ligands. Science. 2013;339: 1426–9. Available: http://www.ncbi.nlm.nih.gov/pubmed/23520111. 10.1126/science.1229159 23520111

[28] Govindaraj RG, Manavalan B, Basith S, Choi S. Comparative analysis of species-specific ligand recognition in Toll-like receptor 8 signaling: a hypothesis. PLoS One. 2011;6: e25118 Available: http://www.pubmedcentral.nih.gov/articlerender.fcgi?artid=3176813&tool=pmcentrez&rendertype=abstract. 10.1371/journal.pone.0025118 21949866 PMC3176813

[29] Yang Z. PAML: a program package for phylogenetic analysis by maximum likelihood. Comput Appl Biosci. 1997;13: 555–6. Available: http://www.ncbi.nlm.nih.gov/pubmed/9367129. 9367129 10.1093/bioinformatics/13.5.555

[30] Yang Z. PAML 4: phylogenetic analysis by maximum likelihood. Mol Biol Evol. 2007;24: 1586–91. Available: http://www.ncbi.nlm.nih.gov/pubmed/17483113. 17483113 10.1093/molbev/msm088

[31] Delport W, Poon AFY, Frost SDW, Kosakovsky Pond SL. Datamonkey 2010: a suite of phylogenetic analysis tools for evolutionary biology. Bioinformatics. 2010;26: 2455–7. Available: http://www.pubmedcentral.nih.gov/articlerender.fcgi?artid=2944195&tool=pmcentrez&rendertype=abstract. 10.1093/bioinformatics/btq429 20671151 PMC2944195

[32] Nielsen R, Yang Z. Likelihood models for detecting positively selected amino acid sites and applications to the HIV-1 envelope gene. Genetics. 1998;148: 929–36. Available: http://www.pubmedcentral.nih.gov/articlerender.fcgi?artid=1460041&tool=pmcentrez&rendertype=abstract. 9539414 10.1093/genetics/148.3.929 PMC1460041

[33] Kosakovsky Pond SL, Frost SDW. Not so different after all: a comparison of methods for detecting amino acid sites under selection. Mol Biol Evol. 2005;22: 1208–22. Available: http://www.ncbi.nlm.nih.gov/pubmed/15703242. 15703242 10.1093/molbev/msi105

[34] Murrell B, Wertheim JO, Moola S, Weighill T, Scheffler K, Kosakovsky Pond SL. Detecting individual sites subject to episodic diversifying selection. PLoS Genet. 2012;8: e1002764 Available: http://www.pubmedcentral.nih.gov/articlerender.fcgi?artid=3395634&tool=pmcentrez&rendertype=abstract. 10.1371/journal.pgen.1002764 22807683 PMC3395634

[35] Niknafs N, Kim D, Kim R, Diekhans M, Ryan M, Stenson PD, et al MuPIT interactive: webserver for mapping variant positions to annotated, interactive 3D structures. Hum Genet. 2013;132: 1235–43. Available: http://www.ncbi.nlm.nih.gov/pubmed/23793516. 10.1007/s00439-013-1325-0 23793516 PMC3797853

[36] Aguileta G, Lengelle J, Marthey S, Chiapello H, Rodolphe F, Gendrault A, et al Finding candidate genes under positive selection in Non-model species: examples of genes involved in host specialization in pathogens. Mol Ecol. 2010;19: 292–306. Available: http://www.ncbi.nlm.nih.gov/pubmed/20041992. 10.1111/j.1365-294X.2009.04454.x 20041992

[37] Areal H, Abrantes J, Esteves PJ. Signatures of positive selection in Toll-like receptor (TLR) genes in mammals. BMC Evol Biol. 2011;11: 368 Available: http://www.pubmedcentral.nih.gov/articlerender.fcgi?artid=3276489&tool=pmcentrez&rendertype=abstract. 10.1186/1471-2148-11-368 22185391 PMC3276489

[38] Barreiro LB, Ben-Ali M, Quach H, Laval G, Patin E, Pickrell JK, et al Evolutionary dynamics of human Toll-like receptors and their different contributions to host defense. PLoS Genet. 2009;5: e1000562 10.1371/journal.pgen.1000562 19609346 PMC2702086

[39] Kim HM, Park BS, Kim J-I, Kim SE, Lee J, Oh SC, et al Crystal structure of the TLR4-MD-2 complex with bound endotoxin antagonist Eritoran. Cell. 2007;130: 906–17. Available: http://www.ncbi.nlm.nih.gov/pubmed/17803912. 17803912 10.1016/j.cell.2007.08.002

[40] Ozinsky A, Underhill DM, Fontenot JD, Hajjar AM, Smith KD, Wilson CB, et al The repertoire for pattern recognition of pathogens by the innate immune system is defined by cooperation between toll-like receptors. Proc Natl Acad Sci U S A. 2000;97: 13766–71. Available: http://www.pubmedcentral.nih.gov/articlerender.fcgi?artid=17650&tool=pmcentrez&rendertype=abstract. 11095740 10.1073/pnas.250476497 PMC17650

[41] Hajjar AM, O’Mahony DS, Ozinsky A, Underhill DM, Aderem A, Klebanoff SJ, et al Cutting edge: functional interactions between toll-like receptor (TLR) 2 and TLR1 or TLR6 in response to phenol-soluble modulin. J Immunol. 2001;166: 15–9. Available: http://www.ncbi.nlm.nih.gov/pubmed/11123271. 11123271 10.4049/jimmunol.166.1.15

[42] Ben-Ali M, Corre B, Manry J, Barreiro LB, Quach H, Boniotto M, et al Functional characterization of naturally occurring genetic variants in the human TLR1-2-6 gene family. Hum Mutat. 2011;32: 643–52. Available: http://www.ncbi.nlm.nih.gov/pubmed/21618349. 10.1002/humu.21486 21618349

[43] Shinkai H, Tanaka M, Morozumi T, Eguchi-Ogawa T, Okumura N, Muneta Y, et al Biased distribution of single nucleotide polymorphisms (SNPs) in porcine Toll-like receptor 1 (TLR1), TLR2, TLR4, TLR5, and TLR6 genes. Immunogenetics. 2006;58: 324–30. Available: http://www.ncbi.nlm.nih.gov/pubmed/16604477. 16604477 10.1007/s00251-005-0068-z

[44] Lipscomb LA, Gassner NC, Snow SD, Eldridge AM, Baase WA, Drew DL, et al Context-dependent protein stabilization by methionine-to-leucine substitution shown in T4 lysozyme. Protein Sci. 1998;7: 765–73. Available: http://www.pubmedcentral.nih.gov/articlerender.fcgi?artid=2143956&tool=pmcentrez&rendertype=abstract. 9541409 10.1002/pro.5560070326 PMC2143956

[45] Kingdon J. The Kingdon field guide to African mammals. San Diego: Academic Press; 1997.

[46] Rietsch A, Beckwith J. The genetics of disulfide bond metabolism. Annu Rev Genet. 1998;32: 163–84. Available: http://www.ncbi.nlm.nih.gov/pubmed/9928478. 9928478 10.1146/annurev.genet.32.1.163

[47] Weber ANR, Morse MA, Gay NJ. Four N-linked glycosylation sites in human toll-like receptor 2 cooperate to direct efficient biosynthesis and secretion. J Biol Chem. 2004;279: 34589–94. Available: http://www.ncbi.nlm.nih.gov/pubmed/15173186. 15173186 10.1074/jbc.M403830200

[48] Lemos de Matos A, McFadden G, Esteves PJ. Positive evolutionary selection on the RIG-I-like receptor genes in mammals. PLoS One. 2013;8: e81864 Available: http://www.pubmedcentral.nih.gov/articlerender.fcgi?artid=3842351&tool=pmcentrez&rendertype=abstract. 10.1371/journal.pone.0081864 24312370 PMC3842351

[49] Smith NGC. Are radical and conservative substitution rates useful statistics in molecular evolution? J Mol Evol. 2003;57: 467–78. Available: http://www.ncbi.nlm.nih.gov/pubmed/14708579. 14708579 10.1007/s00239-003-2500-z

[50] Fowler ME. Husbandry and diseases of captive wild swine and peccaries. Rev Sci Tech. 1996;15: 141–54. Available: http://www.ncbi.nlm.nih.gov/pubmed/8924700. 8924700 10.20506/rst.15.1.913

[51] Patel MR, Loo Y-M, Horner SM, Gale M, Malik HS. Convergent evolution of escape from hepaciviral antagonism in primates. PLoS Biol. 2012;10: e1001282 Available: http://www.pubmedcentral.nih.gov/articlerender.fcgi?artid=3302847&tool=pmcentrez&rendertype=abstract. 10.1371/journal.pbio.1001282 22427742 PMC3302847

[52] Rothenburg S, Seo EJ, Gibbs JS, Dever TE, Dittmar K. Rapid evolution of protein kinase PKR alters sensitivity to viral inhibitors. Nat Struct Mol Biol. 2009;16: 63–70. Available: http://www.pubmedcentral.nih.gov/articlerender.fcgi?artid=3142916&tool=pmcentrez&rendertype=abstract. 10.1038/nsmb.1529 19043413 PMC3142916

