# Supplementary material for: Adaptive Evolution of Toll-Like Receptors (TLRs) in the Family Suidae
Source: PLoS One. 2015 Apr 20;10(4):e0124069. doi: 10.1371/journal.pone.0124069 (PMC4404360; doi:10.1371/journal.pone.0124069)
Supplement: S1 Fig — / ligand binding residues, d residues involved in dimerization, + residues involved in both ligand binding and dimerization. LRR represents leucine rich repeat. LRRNT represents LRR amino termini. LRRCT represents LRR carboxy termini. Human TLR sequences accession numbers: TLR1:Q15399; TLR2:O60603; TLR3: NP_003256.1; TLR6:Q9Y2C9; TLR7:NP_057646; TLR8:NP_619542. Porcine TLR sequences accession numbers: TLR1:NP_001026945; TLR2:NP_998926.1; TLR3:DQ266435.1 TLR6:NP_998925.1; TLR7: DQ647699; TLR8: NP_999352.1. For TLR6, human LRRs were determined from their alignment with murine TLR6. Asterisks, colons and periods under aligned the aligned sequences indicate complete match, strong conservation and weaker conservation of amino acid respectively. (DOCX) [file pone.0124069.s001.docx]

**Figure S1: Domain characterization of TLRs**

LRRNT LRR1

Human_TLR1 MT----SIFHFAIIFMLILQIRIQLSEESEFLVDRSKNGLIHVPKDLSQKTTILNISQNY 56

Porcine_TLR1 MTKENLSIFHFAIIFILILEIRIQLSEESEVLVDRSKTGLTHVPKDLSLETTILDLSQNS 60

** *********:***:**********.******.** ******* :****::***

LRR2 LRR3 LRR4

Human_TLR1 ISELWTSDILSLSKLRILIISHNRIQYLDISVFKFNQELEYLDLSHNKLVKISCHPTVNL 116

Porcine_TLR1 ISELQTSDILSLSKLRVFIISHNRIQYLDVSVFKFNQELEYLDLSHNKLEKI**S**CHP**M**LNL 120

**** ***********::***********:******************* ****** :**

LRR5 LRR6

Human_TLR1 KHLDLSFNAFDALPICKEFGNMSQLKFLGLSTTHLEKSSVLPIAHLNISKVLLVLGETYG 176

Porcine_TLR1 KHLDLSFNAFDALPICQEFGSMFQLEFLGLSATQLQKSSVLPIAHLHIGKVLLVLGDSYG 180

****************:***.* **:*****:*:*:**********:*.*******::**

LRR7 LRR8

Human_TLR1 EKEDPEGLQDFNTESLHIVFPTNKEFHFILDVSVKTVANLELSNIKCVLEDNKCSYFLSI 236

Porcine_TLR1 EREDPESLQDLNTQSLHIVYPPGKEFHFMLDVSVSTAVNLELSNIRCVLDANGCHHFQNV 240

*:****.***:**:*****:*..*****:*****.*..*******:***: * * :* .:

LRR9 / / / LRR10 /

Human_TLR1 LAKLQTNPKLSNLTLNNIETTWNSFIRILQLVWHTTVWYFSISNVKLQGQLDFRDFDYSG 296

Porcine_TLR1 LLKLQKNSKLSNLTLNNIETTWNSFITTLQFVWRTSIEYFSISSVKLQGQLDFRDFDYSD 300

* ***.*.****************** **:**:*:: *****.***************.

LRR11 LRR12 LRR13

/ / d+///+/ /+d / / d/+

Human_TLR1 TSLKALSIHQVVSDVFGFPQSYIYEIFSNMNIKNFTVSGTRMVHMLCPSKISPFLHLDFS 356

Porcine_TLR1 TSLKALSLHQVVSEVFSFPQSYIYKIFSNMNIQYLTVSATHMVHMVCPSQISPFLYLDFS 360

*******:*****:**.*******:*******: :***.*:****:***:*****:****

LRR14 LRR15

d d d d d d

Human_TLR1 NNLLTDTVFENCGHLTELETLILQMNQLKELSKIAEMTTQMKSLQQLDISQNSVSYDEKK 416

Porcine_TLR1 NNALTDMVFKNCANLANLNTLSLQMNQLKELVNVIHMTKEMQSLQQLDVSQNTLRYDENE 420

** *** **:**.:*::*:** ********* :: .**.:*:******:***:: ***::

LRR16 LRR17 LRR18

Human_TLR1 GDCSWTKSLLSLNMSSNILTDTIFRCLPPRIKVLDLHSNKIKSIPKQVVKLEALQELNVA 476

Porcine_TLR1 GSCTWTGSLLSLN**L**SSNILTDSVFRCLPPR**I**KVLDLHNNRIRSIPKDVAHLEALQELNVA 480

*.*:** ******:*******::**************.*:*:****:*.:**********

LRR19 LRR20 LRRCT

Human_TLR1 FNSLTDLPGCGSFSSLSVLIIDHNSVSHPSADFFQSCQKMRSIKAGDNPFQCTCELGEFV 536

Porcine_TLR1 SNSLAHLPGCGSFSSLSILIIDYNSISNPSADFFQSCQKIRSLKAGNNPFQCTCELRDFI 540

***:.***********:****:**:*:***********:**:***:********* :*:

Human_TLR1 KNIDQVSSEVLEGWPDSYKCDYPESYRGTLLKDFHMSELSCNITLLIVTIVATMLVLAVT 596

Porcine_TLR1 QSLGQVSSDVVESWPDSY**E**CEYPESYKGTLLKDFRVSELSCNTALLIVTIGVTGLALALT 600

:.:.****:*:*.*****:*:*****:*******::****** :****** .* *.**:*

Human_TLR1 VTSLCSYLDLPWYLRMVCQWTQTRRRARNIPLEELQRNLQFHAFISYSGHDSFWVKNELL 656

Porcine_TLR1 MTGLCVYFDLPWYLRMLCQWTQTRRRARNVPLEELQRTLQFHAFISYSGHDSAWVKNELL 660

:*.** *:********:************:*******.************** *******

Human_TLR1 PNLEKEGMQICLHERNFVPGKSIVENIITCIEKSYKSIFVLSPNFVQSEWCHYELYFAHH 716

Porcine_TLR1 PNVEKEGIKICLHERNFVPGKSIMENIINCIEKSYKSIFVLSPNFVQSEWCHYELYFAHH 720

**:****::**************:****.*******************************

Human_TLR1 NLFHEGSNSLILILLEPIPQYSIPSSYHKLKSLMARRTYLEWPKEKSKRGLFWANLRAAI 776

Porcine_TLR1 NLFHEGSDNLILILLDSIPQYSIPSSYHKLKALMAQRTYLEWPKEKSKHGLFWANLRASI 780

*******:.******:.**************:***:************:*********:*

Human_TLR1 NIKLTEQAKK------ 786

Porcine_TLR1 NIKLMEKAEEISYTQI 796

**** *:*::

LRRNT LRR1

Human_TLR2 MPHTLWMVWVLGVIISLSKEESSNQAS-LSCDRNGICKGSSGSLNSIPSGLTEAVKSLDL 59

Porcine_TLR2 MPCALWTAWVLGIVISLSKEGAPHQASSLSCDPAGVCDGRSRSLSSIPSGLTAAVKSLDL 60

** :** .****::****** :.:*** **** *:*.* * **.******* *******

LRR2 LRR3

Human_TLR2 SNNRITYISNSDLQRCVNLQALVLTSNGINTIEEDSFSSLGSLEHLDLSYNYLSNLSSSW 119

Porcine_TLR2 SNNRIAYVGSSDLRKCVNLRALRLGANSIHTVEEDSFSSLGSLEHLDLSYNHLSNLSSSW 120

*****:*:..***::****:** * :*.*:*:*******************:********

LRR4 LRR5 LRR6

Human_TLR2 FKPLSSLTFLNLLGNPYKTLGETSLFSHLTKLQILRVGNMDTFTKIQRKDFAGLTFLEEL 179

Porcine_TLR2 FKSLSTLKFLNLLGNPYKTLGEAPLFSHLPNLRILKIGNNDTFPEIQAKDFQGLTFLQEL 180

**.**:*.**************:.*****.:*:**::** ***.:** *** *****:**

LRR7 LRR8

Human_TLR2 EIDASDLQSYEPKSLKSIQNVSHLILHMKQHILLLEIFVDVTSSVECLELRDTDLDTFHF 239

Porcine_TLR2 EIGASHLQRYAPKSLRSIQNISHLILHMRRPALLPKIFVDLLSSLEYLELRNTDFSTF**N**F 240

**.**.** * ****:****:*******:: ** :****: **:* ****:**:.**:*

LRR9 / / LRR10 / / / //

Human_TLR2 SELSTGETNSLIKKFTFRNVKITDESLFQVMKLLNQISGLLELEFDDCTLNGVGNFRASD 299

Porcine_TLR2 SDVSINEHCTVMKKFTFRKAEITDASFTEIVKLLNYVSGALEVEFDDCTLNGRGDLSTSA 300

*::* .* :::******:.:*** *: :::**** :** **:********* *:: :*

LRR11 LRR12

/ / / /d/ ddd+// / / / /d d/++/+ /

Human_TLR2 NDRVIDPGKVETLTIRRLHIPRFYLFYDLSTLYSLTERVKRITVENSKVFLVPCLLSQHL 359

Porcine_TLR2 LDTIKSLGNVETLTVRRLHIPQFFLFYDLRSIYSLTG**A**VKRITIENSKVFLVPCSLSQHL 360

* : . *:*****:******:*:***** ::**** *****:********** *****

LRR13 d d dddd d LRR14 d LRR15

Human_TLR2 KSLEYLDLSENLMVEEYLKNSACEDAWPSLQTLILRQNHLASLEKTGETLLTLKNLTNID 419

Porcine_TLR2 KSLEYLDLSENLMSEEYLKNSACEHAWPFLHTLILRQNHLKSLEKTGEVLVTLKNLTNLD 420

************* **********.*** *:********* *******.*:*******:*

LRR16 LRR17 LRR18

Human_TLR2 ISKNSFHSMPETCQWPEKMKYLNLSSTRIHSVTGCIPKTLEILDVSNNNLNLFSLNLPQL 479

Porcine_TLR2 ISKNNFDSMPETCQWPEKMKYLNLSSTRIHSLTHCLPQTLEVLDISNNNLNSFSLSLPQL 480

****.*.************************:* *:*:***:**:****** ***.****

LRR19 LRR20

Human_TLR2 KELYISRNKLMTLPDASLLPMLLVLKISRNAITTFSKEQLDSFHTLKTLEAGGNNFICSC 539

Porcine_TLR2 KELYISRNKLKTLPDASFLPMLSVLRISRNTINTFSKEQLDSFQKLKTLEAGGNNFICSC 540

********** ******:**** **:****:*.**********:.***************

LRRCT

Human_TLR2 EFLSFTQEQQALAKVLIDWPANYLCDSPSHVRGQQVQDVRLSVSECHRTALVSGMCCALF 599

Porcine_TLR2 DFLSFTQGQQALAQVLSDWPENYLCDSPSHVRGQRVQDTRLSLTECHRVAVVSVVCCALF 600

:****** *****:** *** *************:***.***::****.*:** :*****

Human_TLR2 LLILLTGVLCHRFHGLWYMKMMWAWLQAKRKPRKAPSRNICYDAFVSYSERDAYWVENLM 659

Porcine_TLR2 LLLLLTGALCHHFHGLWCMKMMWAWLQAKRKPRKAPRRDVCYDAFVSYSEQDSYWVENLM 660

**:****.***:***** ****************** *::**********:*:*******

Human_TLR2 VQELENFNPPFKLCLHKRDFIPGKWIIDNIIDSIEKSHKTVFVLSENFVKSEWCKYELDF 719

Porcine_TLR2 VQELEHFQPPFKLCLHKRDFIPGKWIIDNIIDSIEKSQKTIFVLSENFVKSEWCKYELDF 720

*****:*:*****************************:**:*******************

Human_TLR2 SHFRLFDENNDAAILILLEPIEKKAIPQRFCKLRKIMNTKTYLEWPMDEAQREGFWVNLR 779

Porcine_TLR2 SHFRLFDENDDTAILILLEPIEKKTIPQRFCKLRKIMNTRTYLEWPADETQREGFWLNLR 780

*********:*:************:**************:****** **:******:***

Human_TLR2 AAIKS 784

Porcine_TLR2 AAIKS 785

LRRNT/ LRR1

Human_TLR3 MRQTLPC-IYFWGGLLPFGMLCASSTTKCTVSHEVADCSHLKLTQVPDDLPTNITVLNLT 59

Porcine_TLR3 MSRSLPCHIYSFWVLLPFWILYTTSTNKCTVRHEIADCSHLKLTQIPDDLPANITVLNLT 60

* ::*** ** : **** :* ::**.**** **:**********:*****:********

LRR2 LRR3

/ / / / / / / /

Human_TLR3 HNQLRRLPAANFTRYSQLTSLDVGFNTISKLEPELCQKLPMLKVLNLQHNELSQLSDKTF 119

Porcine_TLR3 HNQLRGLPPANFTIYSQLTTLDGGFNTIPKLEPELCQSLPLLDILNLQHNELSQLSDKTF 120

***** **.**** *****:** *****.********.**:*.:****************

LRR4 LRR5 LRR6

Human_TLR3 AFCTNLTELHLMSNSIQKIKNNPFVKQKNLITLDLSHNGLSSTKLGTQVQLENLQELLLS 179

Porcine_TLR3 IFCMNLIELHLMSNSIQKIQNNPFKNLKNLIKLDLSHNGLSSTKLGTQLQLENLQELLLA 180

** ** ************:**** : ****.****************:**********:

LRR7 LRR8

Human_TLR3 NNKIQALKSEELDIFANSSLKKLELSSNQIKEFSPGCFHAIGRLFGLFLNNVQLGPSLTE 239

Porcine_TLR3 NNKISALKREELDFLGNSSLKRLELSSNQIQEFSPGCFHAIGKLFGLSLNNVKLSPSLTE 240

****.*** ****::.*****:********:***********:**** ****:*.*****

LRR9 LRR10

Human_TLR3 KLCLELANTSIRNLSLSNSQLSTTSNTTFLGLKWTNLTMLDLSYNNLNVVGNDSFAWLPQ 299

Porcine_TLR3 KLCLELSNTSIENLSLSNIQLYKTSNTTFFGLKQTNLSMLDLSHNSLSVIGNDSFAWLPH 300

******:****.****** ** .******:*** ***:*****:*.*.*:*********:

LRR11 LRR12

Human_TLR3 LEYFFLEYNNIQHLFSHSLHGLFNVRYLNLKRSFTKQSISLASLPKIDDFSFQWLKCLEH 359

Porcine_TLR3 LKYFFLEYNNIERLSSRSLYGLSNVKYLNLRRSFTKQSISLASLPKIEDFSFQWLKSLEY 360

*:*********::* *:**:** **:****:****************:********.**:

LRR13 LRR14 LRR15

Human_TLR3 LNMEDNDIPGIKSNMFTGLINLKYLSLSNSFTSLRTLTNETFVSLAHSPLHILNLTKNKI 419

Porcine_TLR3 LNMEDNNFPGIKRNTFTGLIKLKSLSLSNSFSSLRTLTNETFISLADSPLLILNLTKNKI 420

******::**** * *****:** *******:**********:***.*** *********

LRR16 LRR17

Human_TLR3 SKIESDAFSWLGHLEVLDLGLNEIGQELTGQEWRGLENIFEIYLSYNKYLQLTRNSFALV 479

Porcine_TLR3 SKIESGAFSWLGHLKVLDLGLNEIGQELTGQEWRGLKNIVEVYLSYNRYLELTTDSFALV 480

*****.********:*********************:**.*:*****:**:** :*****

LRR18 / / LRR19 LRR20 /

Human_TLR3 PSLQRLMLRRVALKNVDSSPSPFQPLRNLTILDLSNNNIANINDDMLEGLEKLEILDLQH 539

Porcine_TLR3 PSLQQLMLRRVALRDMDCSPSPFHPLFNLTILDLSNNNIANINDELLKGLEKLQILDLQH 540

****:********:::*.*****:** *****************::*:*****:******

/ / / LRR21 LRR22

Human_TLR3 NNLARLWKHANPGGPIYFLKGLSHLHILNLESNGFDEIPVEVFKDLFELKIIDLGLNNLN 599

Porcine_TLR3 NNLARLWKHANPGGPVQFLKGLSHLHILNLESNGFDEIPADAFRDLSELKSIDLGLNNLN 600

***************: **********************.:.*:** *** *********

/ LRR23 LRRCT

Human_TLR3 TLPASVFNNQVSLKSLNLQKNLITSVEKKVFGPAFRNLTELDMRFNPFDCTCESIAWFVN 659

Porcine_TLR3 ILPPSVFDNQVSLKSLSLQKNLITSVKKTVFGPAFQKLSNLDMRFNPFDCTCESIAWFVS 660

**.***:********.*********:*.******::*::*******************.

Human_TLR3 WINETHTNIPELSSHYLCNTPPHYHGFPVRLFDTSSCKDSAPFELFFMINTSILLIFIFI 719

Porcine_TLR3 WINSTHTNISELSSHYLCNTPPQYHGLPVILFDTSPCKDSAPFELFFMITASMLLIFIFI 720

***.*****.************:***:** *****.*************.:*:*******

Human_TLR3 VLLIHFEGWRISFYWNVSVHRVLGFKEIDRQTEQFEYAAYIIHAYKDKDWVWEHFSSMEK 779

Porcine_TLR3 ILLIHFEGWRISFYWNVSVHRVLGFKEIDKQPEQFEYAAYIIHAYKDRDWVWEHFAPMEE 780

:****************************:*.***************:*******:.**:

Human_TLR3 EDQSLKFCLEERDFEAGVFELEAIVNSIKRSRKIIFVITHHLLKDPLCKRFKVHHAVQQA 839

Porcine_TLR3 KDETLRFCLEERDFEAGALELEAIVNSIKRSRKIIFVITQHLLKDPLCKRFKVHHAVQQA 840

:*::*:***********.:********************:********************

Human_TLR3 IEQNLDSIILVFLEEIPDYKLNHALCLRRGMFKSHCILNWPVQKERIGAFRHKLQVALGS 899

Porcine_TLR3 IEQNLDSIILIFLEEIPDYKLNHALCLRRGMFKSHCILNWPVQKERINAFHHKLQVALGS 900

**********:************************************.**:*********

Human_TLR3 KNSVH 904

Porcine_TLR3 RNSVH 905

:****

LRRNT LRR1

Human_TLR6 MTKDKEPIVKSFHFVCLMIIIVGTRIQFSDGNEFAVDKSKRGLIHVPKDLPLKTKVLDMS 60

Porcine_TLR6 MTKDKKPTVISLHSVYVMTLVWGTLIQFSEESEFVVDKSKIGLTRVPKDLPPQTKVLDVS 60

*****:* * *:* * :* :: ** ****: .**.***** ** :****** :*****:*

LRR2 LRR3

Human_TLR6 QNYIAELQVSDMSFLSELTVLRLSHNRIQLLDLSVFKFNQDLEYLDLSHNQLQKISCHPI 120

Porcine_TLR6 QNFITELHLSDISFLSQLTVLRLSQNRMQCLDISVFKFNQDLEYLDLSHNQLQTILCHPI 120

**:*:**::**:****:*******:**:* **:********************.* ****

LRR4 LRR5 LRR6

Human_TLR6 VSFRHLDLSFNDFKALPICKEFGNLSQLNFLGLSAMKLQKLDLLPIAHLHLSYILLDLRN 180

Porcine_TLR6 TSLKHLDLSFNDFEALPICKEFGNLTQLNFLGLSATKLQQLDLLPIAHLHLSCILLDLER 180

.*::*********:***********:********* ***:************ *****..

LRR7 LRR8

Human_TLR6 YYIKENETESLQILNAKTLHLVFHPTSLFAIQVNISVNTLGCLQLTNIKLNDDNCQVFIK 240

Porcine_TLR6 YY**M**KENEKESLQILNTEKLHLVFHPNSFFSVQVNISVKSVGCLQLANIKLGDDNCQVFIT 240

**:****.*******::.*******.*:*::******:::*****:****.********.

LRR9 LRR10

Human_TLR6 FLSELTRGSTLLNFTLNHIETTWKCLVRVFQFLWPKPVEYLNIYNLTIIESIREEDFTYS 300

Porcine_TLR6 FLLELTQGPTLLNFTLNHVETTWKCLVGIFQFLWPKPVEYLSIYNLTIVESIDEEDFIYY 300

** ***:*.*********:******** :************.******:*** **** *

LRR11 LRR12 LRR13

d dd d+/ d d d d d

Human_TLR6 KTTLKALTIEHITNQVFLFSQTALYTVFSEMNIMMLTISDTPFIHMLCPHAPSTFKFLNF 360

Porcine_TLR6 ETTLKG**V**KIEHITKRVFIFSQTALYRVFSDMNI**R**MLTIADTHFIHMLCPQVPSTFNFLNF 360

:****.:.*****::**:******* ***:*** ****:** *******:.****:****

d d dd d LRR14 LRR15

Human_TLR6 TQNVFTDSIFEKCSTLVKLETLILQKNGLKDLFKVGLMTKDMPSLEILDVSWNSLESGRH 420

Porcine_TLR6 TQNVFTDSVFQNCKTLARLETLILQKNKLEDLFKISLMTKDMLSLEILDVSSNSLEYDRH 420

********:*::*.**.:********* *:****:.****** ******** **** .**

LRR16 LRR17 LRR18

Human_TLR6 KENCTWVESIVVLNLSSNMLTDSVFRCLPPRIKVLDLHSNKIKSVPKQVVKLEALQELNV 480

Porcine_TLR6 GENCTWVGSIVVLNLSSNILTDSVFRCL**P**PRIKVLDLHSNRIRSIPKDVAHLEALQELNV 480

****** **********:*********************:*:*:**:*.:*********

LRR19 LRR20 LRRCT

Human_TLR6 AFNSLTDLPGCGSFSSLSVLIIDHNSVSHPSADFFQSCQKMRSIKAGDNPFQCTCELREF 540

Porcine_TLR6 ASNSLAHLPGCGSFSSLSIL**S**IDYNSISNPSADFFQSCQKIRSLKAGNNPFQCTCELRDF 540

* ***:.***********:* **:**:*:***********:**:***:**********:*

Human_TLR6 VKNIDQVSSEVLEGWPDSYKCDYPESYRGSPLKDFHMSELSCNITLLIVTIGATMLVLAV 600

Porcine_TLR6 IQSLGQVSSDVVE**S**WPDSY**E**CEYPESYKGTLLKDFRVSELSCNTALLIVTIGVTGLALAL 600

::.:.****:*:*.*****:*:*****:*: ****::****** :*******.* *.**:

Human_TLR6 TVTSLCIYLDLPWYLRMVCQWTQTRRRARNIPLEELQRNLQFHAFISYSEHDSAWVKSEL 660

Porcine_TLR6 TMTGLCVYFDLPWYLRMLCQWTQTRRRARNVPLEELQRTLQFHAFISYSEHDSAWVKNEL 660

*:*.**:*:********:************:*******.******************.**

Human_TLR6 VPYLEKEDIQICLHERNFVPGKSIVENIINCIEKSYKSIFVLSPNFVQSEWCHYELYFAH 720

Porcine_TLR6 VPCLEKEGIKICLHERNFVPGKSIMENIINCIEKSYKSIFVLSPNFVQSEWCHYELYFAH 720

** ****.*:**************:***********************************

Human_TLR6 HNLFHEGSNNLILILLEPIPQNSIPNKYHKLKALMTQRTYLQWPKEKSKRGLFWANIRAA 780

Porcine_TLR6 HNLFHEGSDNLILILLDPIPQNSIPGKYHKLKALMAQRTYLEWPKEKSKHGPFWANIRAA 780

********:*******:********.*********:*****:*******:* ********

Human_TLR6 FNMKLTLVTENNDVKS 796

Porcine_TLR6 FNIKLKLVAEEDDVKT 796

**:**.**:*::***:

LRRNT

Human_TLR7 MVFPMWTLKRQILILFNIILISKLLGARWFPKTLPCDVTLDVPKNHVIVDCTDKHLTEIP 60

Porcine_TLR7 -VFPMWTLKRQFLILFNIVLISELLGARWFPKTLPCDVSLDAPNAHVIVDCTDKHLTAIP 59

**********:******:***:***************:**.*: ************ **

LRR1 LRR2

Human_TLR7 GGIPTNTTNLTLTINHIPDISPASFHRLDHLVEIDFRCNCVPIPLGSKNNMCIKRLQIKP 120

Porcine_TLR7 GGIPTNATNLTLTINHIASITPASFQQLDHLVEIDFRCNCIPVRLGPKDNLCTRRQQIKP 119

******:**********..*:****::*************:*: **.*:*:* :* ****

LRR3 LRR4 LRR5

Human_TLR7 RSFSGLTYLKSLYLDGNQLLEIPQGLPPSLQLLSLEANNIFSIRKENLTELANIEILYLG 180

Porcine_TLR7 SSFSKLTYLKALYLDGNQLLEIPRDLPPSLQLLSLEANNIFWIMKENLTELANLEMLYLG 179

*** *****:************:.**************** * *********:*:****

LRR6 LRR7

Human_TLR7 QNCYYRNPCYVSYSIEKDAFLNLTKLKVLSLKDNNVTAVPTVLPSTLTELYLYNNMIAKI 240

Porcine_TLR7 QNCYYRNPCNVSFSIEKDAFLSLRNLKLLSLKDNNISAVPTVLPSTLTELFLYNNIIAKI 239

********* **:********.* :**:*******::*************:****:****

LRR8 LRR9

Human_TLR7 QEDDFNNLNQLQILDLSGNCPRCYNAPFPCAPCKNNSPLQIPVNAFDALTELKVLRLHSN 300

Porcine_TLR7 QEDDFNNLSQPQVLDLSGNCPRCYNVPFPCTPCENNAPLQIHLHAFDALTELQVLRLHSN 299

********.* *:************.****:**:**:**** ::********:*******

LRR10 LRR11

Human_TLR7 SLQHVPPRWFKNINKLQELDLSQNFLAKEIGDAKFLHFLPSLIQLDLSFNFELQVYRASM 360

Porcine_TLR7 SLQYVPQRWFQNLNKLKELDLSQNFLAKEIGDAKFLHLLPNLVKLDLSFNYELQVYHTFM 359

***:** ***:*:***:********************:**.*::******:*****:: *

LRR12 LRR13

Human_TLR7 NLSQAFSSLKSLKILRIRGYVFKELKSFNLSPLHNLQNLEVLDLGTNFIKIANLSMFKQF 420

Porcine_TLR7 NLSDSFSSLKNLKVLRIKGYVFKELKSLNLSPLRNLPNLEVLDLGTNFIKIANLSIFKQF 419

***::*****.**:***:*********:*****:** ******************:****

LRR14

Human_TLR7 KRLKVIDLSVNKISPSGDSSEVGFCSNARTSVESYEPQVLEQLHYFRYDKYARSCRFKNK 480

Porcine_TLR7 KTLKFIDLSVNKISPSGDSSESGFCSG**M**RTSAESHGPQVLESLHYFRYDEYARSCRFKNK 479

* **.**************** ****. ***.**: *****.*******:**********

/ / LRR15 / / LRR16

Human_TLR7 E-ASFMSVNESCYKYGQTLDLSKNSIFFVKSSDFQHLSFLKCLNLSGNLISQTLNGSEFQ 539

Porcine_TLR7 EPSSSLPLNEDCSMYGQTLDLSRNNIFFIRSSEFQHLTFLKCLNLSGNSISQALNGSEFQ 539

* :* :.:**.* ********:*.***::**:****:********** ***:*******

/ / / LRR17 / / LRR18

Human_TLR7 PLAELRYLDFSNNRLDLLHSTAFEELHKLEVLDISSNSHYFQSEGITHMLNFTKNLKVLQ 599

Porcine_TLR7 PLVELKYLDFSNNRLDLLHSTAFEELRNLEVLDISSNSHYFQSEGITHMLDFTKNLKVLK 599

**.**:********************::**********************:********:

LRR19 LRR20 LRR21

Human_TLR7 KLMMNDNDISSSTSRTMESESLRTLEFRGNHLDVLWREGDNRYLQLFKNLLKLEELDISK 659

Porcine_TLR7 KLMMNNNDIATSTSTTMESESLRILEFRGNHLDILWRDGDNRYLKFFKNLHKLEELDISE 659

*****:***::*** ******** *********:***:******::**** ********:

LRR22 LRR23

Human_TLR7 NSLSFLPSGVFDGMPPNLKNLSLAKNGLKSFSWKKLQCLKNLETLDLSHNQLTTVPERLS 719

Porcine_TLR7 NSLSFLPSGVFDGMPPNLKTLSLAKNGLKSFNWGKLQYLQNLETLDLSYNQLKTVPERLS 719

*******************.***********.* *** *:********:***.*******

LRR24 LRR25

Human_TLR7 NCSRSLKNLILKNNQIRSLTKYFLQDAFQLRYLDLSSNKIQMIQKTSFPENVLNNLKMLL 779

Porcine_TLR7 NCSRSLKKLILKNNEIRNLTKYFLQDAFQLRHLDLSSNKIQVTQKTSFPENVLNNLQILF 779

*******:******:**.*************:*********: *************::*:

LRR26 LRRCT

Human_TLR7 LHHNRFLCTCDAVWFVWWVNHTEVTIPYLATDVTCVGPGAHKGQSVISLDLYTCELDLTN 839

Porcine_TLR7 LHHNRFLCNCDAVWLVWWVNHTEVTIPFLATDVTCMGPGAHKGQSVVSLDLYTCELDLTN 839

********.*****:************:*******:**********:*************

Human_TLR7 LILFSLSISVSLFLMVMMTASHLYFWDVWYIYHFCKAKIKGYQRLISPDCCYDAFIVYDT 899

Porcine_TLR7 FVLFSLSLSAVLFLIVITIANHLYFWDVWYSYHFCKAKIKGYQRLISPNSCYDAFIVYDT 899

::*****:*. ***:*: *.********* *****************:.**********

Human_TLR7 KDPAVTEWVLAELVAKLEDPREKHFNLCLEERDWLPGQPVLENLSQSIQLSKKTVFVMTD 959

Porcine_TLR7 KDPAVTEWVLDELVAKLEDPREKHFNLCLEERDWLPGQPVLENLSQSIQLSKKTVFVMTD 959

********** *************************************************

Human_TLR7 KYAKTENFKIAFYLSHQRLMDEKVDVIILIFLEKPFQKSKFLQLRKRLCGSSVLEWPTNP 1019

Porcine_TLR7 KYAKTEKFKIAFYLSHQRLMDEKVDVIILIFLEKPLQKSKFFQLRKRLCGSSVLEWPTNP 1019

******:****************************:*****:******************

Human_TLR7 QAHPYFWQCLKNALATDNHVAYSQVFKETV 1049

Porcine_TLR7 QAHPYFWQCLKNALATDNHVTYSQVFKETA 1049

********************:********.

LRRNT

Human_TLR8 MENMFLQSSMLTCIFLLISGSCELCAEENFSRSYPCDEKKQNDSVIAECSNRRLQEVPQT 60

Porcine_TLR8 ---MTLHFLLLTCLFLLIPDSCEFFTGANYSRSYPCDERKENGSVIAECNNRQLQEVPRR 57

* *: :***:****..***: : *:********:*:*.******.**:*****:

LRR1 LRR2

Human_TLR8 VGKYVTELDLSDNFITHITNESFQGLQNLTKINLNHNPNVQHQNGNPGIQSNGLNITDGA 120

Porcine_TLR8 VGNYVTELDLSDNFIRRITNESFQGLQNLTKINLNHNAKLWPQS------ENGMTITDGA 111

**:************ :********************.:: *. .**:.*****

LRR3 LRR4 LRR5

Human_TLR8 FLNLKNLRELLLEDNQLPQIPSGLPESLTELSLIQNNIYNITKEGISRLINLKNLYLAWN 180

Porcine_TLR8 FLNLHHLRELLLEDNQLREIPTGLPESLRELSLIQNKIILLNTKNMFGLRKLESLYLGWN 171

****::*********** :**:****** *******:* :..:.: * :*:.***.**

dddd LRR6 LRR7

Human_TLR8 CYFNKVCEKTNIEDGVFETLTNLELLSLSFNSLSHVPPKLPSSLRKLFLSNTQIKYISEE 240

Porcine_TLR8 CYFT-CNETFIIDEGAFENLTNLKVLSLSFNTLYRVPPKLPSSLTKLYLSNTKIRNINQE 230

***. *. *::*.**.****::******:* :********* **:****:*: *.:*

dddddd LRR8 LRR9

Human_TLR8 DFKGLINLTLLDLSGNCPRCFNAPFPCVPCDGGASINIDRFAFQNLTQLRYLNLSSTSLR 300

Porcine_TLR8 DFKGLENLRVLDLSGNCPRCFNAPFPCNPCPGDASIQIHPLAFRYLTELRYLNLSSTSLR 290

***** ** :***************** ** *.***:*. :**: **:************

LRR10 / / / LRR11

Human_TLR8 KINAAWFKNMPHLKVLDLEFNYLVGEIASGAFLTMLPRLEILDLSFNYIKGSYPQHINIS 360

Porcine_TLR8 RIPATWFENLHHLKVLHLEFNYLMDEIASGEFLAKLPSLEILDLSYNYEQKKYPQYINIS 350

:* *:**:*: *****.******:.***** **: ** *******:** : .***:****

/ / LRR12 / / LRR13

Human_TLR8 RNFSKLLSLRALHLRGYVFQELREDDFQPLMQLPNLSTINLGINFIKQIDFKLFQNFSNL 420

Porcine_TLR8 HYFANLTSLQILHLRAYVFQELRKEDFQPLRNLLHL**KF**INLGINFIKQIDFTIF**S**EFSNL 410

: *::* **: ****.*******::***** :* :*. *************.:*.:****

dd/ ddd LRR14

Human_TLR8 EIIYLSENRISPLVKDTRQSYANSSSFQRHIRKRRSTDFEFDPHSNFYHFTRPLIKPQCA 480

Porcine_TLR8 SIIYLSENRISPLVNNTGQKNGDRPSFQSHVLKPRSATPKFDPHSNFYHNTKPLIKPQCS 470

.*************::* *. .: .*** *: * **: :********* *:*******:

ddd d LRR15 LRR16 ddd / LRR17

Human_TLR8 AYGKALDLSLNSIFFIGPNQFENLPDIACLNLSANSNAQVLSGTEFSAIPHVKYLDLTNN 540

Porcine_TLR8 RYGKALDLSLNSIFFIGPNQFEAFKDIACLNLSSNGNGQVLHGSEFSHLPGIKYLDLTNN 530

********************* : ********:*.*.*** *:*** :* :********

d / / ddd ddd/// LRR18 LRR19 dddd

Human_TLR8 RLDFDNASALTELSDLEVLDLSYNSHYFRIAGVTHHLEFIQNFTNLKVLNLSHNNIYTLT 600

Porcine_TLR8 RLDFDDDAAFSELPLLEVLDLSYNSHYFRIAGVTHRLGFIQNLPQLRVLNLSHNSIFTLT 590

*****: :*::**. ********************:* ****:.:*:*******.*:***

d LRR20 d dddddd LRR21 d

Human_TLR8 DKYNLESKSLVELVFSGNRLDILWNDDDNRYISIFKGLKNLTRLDLSLNRLKHIPNEAFL 660

Porcine_TLR8 ETY-LKSTSLKELVFSGNRLDLLWNAQDDRYWQIFKNLSTLTHLDLSSNNLQHIPSEAFL 649

:.* *:*.** **********:*** :*:** .***.*..**:**** *.*:***.****

LRR22 LRR23 LRR24

Human_TLR8 NLPASLTELHINDNMLKFFNWTLLQQFPRLELLDLRGNKLLFLTDSLSDFTSSLRTLLLS 720

Porcine_TLR8 NLPQTLTELYISDNRLNFFNWSLLQQFPNLTLLDLSGNELSFLTDSLSKFTTSLQTLILR 709

*** :****:*.** *:****:******.* **** **:* *******.**:**:**:*

LRR25 LRR26

Human_TLR8 HNRISHLPSGFLSEVSSLKHLDLSSNLLKTINKSALETKTTTKLSMLELHGNPFECTCDI 780

Porcine_TLR8 QNRISYLPSGLLSEASSLTHLDLSSNQLKM**V**NISKLHAKTTTNLAILKLDRNPFDCTCDI 769

:****:****:***.***.******* ** :* * *.:****:*::*:*. ***:*****

LRRCT

Human_TLR8 GDFRRWMDEHLNVKIPRLVDVICASPGDQRGKSIVSLELTTCVSDVTAVILFFFTFFITT 840

Porcine_TLR8 RDFRKWMD**E**NLKVTIPRLTDVICASPGDQRGRSIVSLELTTCVSDTIAAIICFFTFFVTS 829

***:****:*:*.****.************:*************. *.*: *****:*:

Human_TLR8 MVMLAALAHHLFYWDVWFIYNVCLAKVKGYRSLSTSQTFYDAYISYDTKDASVTDWVINE 900

Porcine_TLR8 TVMLAALAHHWFYWDAWFIYHVCLAKVKGYRSLPTSQTFYDAYVSYDTKDASVTDWVMNE 889

********* ****.****:************.*********:*************:**

Human_TLR8 LRYHLEESRDKNVLLCLEERDWDPGLAIIDNLMQSINQSKKTVFVLTKKYAKSWNFKTAF 960

Porcine_TLR8 LRFHLEESEGKNVLLCLEERDWDPGLAIIDNLMQSINQSKKTIFVLTKKYAKNWNFKTAF 949

**:*****..********************************:*********.*******

Human_TLR8 YLALQRLMDENMDVIIFILLEPVLQHSQYLRLRQRICKSSILQWPDNPKAEGLFWQTLRN 1020

Porcine_TLR8 YLALQRLMDENMDVIVFILLEPVLQHSQYLRLRQRICKSSILQWPDNPKAEGLFWQSLKN 1009

***************:****************************************:*:*

Human_TLR8 VVLTENDSRYNNMYVDSIKQY 1041

Porcine_TLR8 VVLTENDSRYNSLYVNSIK-- 1028

***********.:**:***

/ ligand binding residues, d residues involved in dimerization, + residues involved in both ligand binding and dimerization. LRR represents leucine rich repeat. LRRNT represents LRR amino termini. LRRCT represents LRR carboxy termini. Human TLR sequences accession numbers: TLR1:Q15399; TLR2:O60603; TLR3: NP_003256.1; TLR6:Q9Y2C9; TLR7: NP_057646; TLR8:NP_619542. Porcine TLR sequences accession numbers: TLR1:NP_001026945; TLR2:NP_998926.1; TLR3:DQ266435.1 TLR6:NP_998925.1; TLR7: DQ647699; TLR8: NP_999352.1. For TLR6, human LRRs were determined from their alignment with murine TLR6. Asterisks, colons and periods under aligned the aligned sequences indicate complete match, strong conservation and weaker conservation of amino acid respectively.
