# Supplementary material for: Adaptive Evolution of Toll-Like Receptors (TLRs) in the Family Suidae
Source: PLoS One. 2015 Apr 20;10(4):e0124069. doi: 10.1371/journal.pone.0124069 (PMC4404360; doi:10.1371/journal.pone.0124069)
Supplement: S1 Table — aExon encoding the extracellular domain. (DOCX) [file pone.0124069.s003.docx]

**Table S1.** Summary of TLR extracellular domains studied

| **Gene** | **Exon no^a^** | **Genomic coordinates of extracellular domain^b^** | **Aligned length (bp) of extracellular domain sequences^c^** |
| --- | --- | --- | --- |
| *TLR1* | 3 | 8:31628613-31630280:-1 | 1668 |
| *TLR2* | 2 | 8:79825324-79827018:-1 | 1695 |
| *TLR3* | 4 | 15:53849144-53849505:-1 | 366 |
| *TLR3* | 5 | 15:53848182-53848373:-1 | 192 |
| *TLR3* | 6 | 15:53841845-53843311:-1 | 1467 |
| *TLR6* | 2 | 8:31642930-31644612:-1 | 1683 |
| *TLR7* | 2 | X:10472926-10475370:1 | 2445 |
| *TLR8* | 4 | X:10509874-10512249:1 | 2376 |

**^a^**Exon encoding the extracellular domain

**^b^**Genomic coordinates were determined by blat search of porcine TLR mRNA sequences against *Sus scrofa* genome assembly build 10.2.

**^c^**Sample origin for species for which sequences were obtained are indicated in bracket as follows: *Potomochoerus larvatus* (San diego zoo, USA), *Potomochoerus porcus* (San diego zoo, USA), *Phacochoerus africanus* (Omaha’s zoo, USA), *Babyrousa Babyrousa* (San diego zoo, USA), *Sus verucossus* (Surabaya zoo, Indonesia), *Sus celebensis* (San diego zoo, USA), *Sus barbatus* (Omaha’s zoo, USA), *Sus cebifrons* (San diego zoo, USA), *Sus scrofa* ,Europe, (Meinweg, Roerdalen, Netherlands), *Sus* *scrofa*, Asia (North China).
