# Supplementary material for: Adaptive Evolution of Toll-Like Receptors (TLRs) in the Family Suidae
Source: PLoS One. 2015 Apr 20;10(4):e0124069. doi: 10.1371/journal.pone.0124069 (PMC4404360; doi:10.1371/journal.pone.0124069)
Supplement: S2 Table — (DOCX) [file pone.0124069.s004.docx]

**Table S2.** Results from site models at codons under persistent positive selection in members of the family Suidae

| **Gene** | **Codons** | **Tests of selection** | | | | | |
| --- | --- | --- | --- | --- | --- | --- | --- |
|  |  | **PAML 2a** | | **PAML M8** | | **FEL**  **p < 0.1** | **REL**  **BF > 50** |
|  |  | pp > 0.95 | 2ln∆L^a^ | pp > 0.95 | 2ln∆L^b^ |  |  |
| *TLR1* | 117 | 0.9850 | 21.3890** | 0.9940 | 21.6814** |  | 167.5190 |
|  | 434 | 0.9970 |  | 0.9990 |  |  | 5232.9900 |
|  | 451 |  |  | 0.9610 |  |  | 58.204 |
|  | 559 | 0.9930 |  | 0.9970 |  |  | 8417.3400 |
| *TLR2* | 216 | 0.9580 | 7.8286** | 0.9800 | 8.0464** |  | 354.448 |
|  | 338 | 0.9620 |  | 0.9900 |  |  | 370.5550 |
| *TLR6* | 49 |  |  |  |  |  | 98.3187 |
|  | 63 |  |  |  |  |  | 107.5270 |
|  | 79 |  |  |  |  |  | 84.4861 |
|  | 121 |  |  |  |  |  | 80.5618 |
|  | 180 |  |  |  |  |  | 84.6772 |
|  | 183 |  | 16.1352 | 0.9520 | 16.1358** |  | 742.6930 |
|  | 187 |  |  |  |  |  | 97.7689 |
|  | 213 |  |  |  |  |  | 92.1804 |
|  | 269 |  |  |  |  |  | 98.6309 |
|  | 307 |  |  |  |  |  | 137.7390 |
|  | 334 |  |  | 0.9660 |  |  | 1210.1800 |
|  | 356 |  |  |  |  |  | 104.0040 |
|  | 386 |  |  |  |  |  | 102.0440 |
|  | 394 |  |  |  |  |  | 98.9399 |
|  | 452 | 0.9880 |  | 0.9960 |  | 0.0704 | 1844.8500 |
|  | 459 |  |  | 0.9540 |  |  | 922.0600 |
|  | 467 |  |  |  |  |  | 102.7000 |
|  | 470 |  |  |  |  |  | 79.1197 |
|  | 501 |  |  | 0.9610 |  |  | 1592.2300 |
|  | 536 |  |  |  |  |  | 92.1069 |
|  | 554 |  |  | 0.9560 |  |  | 1087.2700 |
|  | 560 |  |  | 0.9650 |  |  | 1104.8600 |
| *TLR8* | 178 |  | 4.6024 |  | 5.0782 |  | 226.4190 |
|  | 236 |  |  |  |  |  | 232.4120 |
|  | 387 |  |  |  |  |  | 260.8170 |
|  | 388 |  |  | 0.9590 |  |  | 711.3420 |
|  | 405 |  |  |  |  |  | 300.8590 |
|  | 412 |  |  |  |  |  | 258.9650 |
|  | 740 |  |  |  |  |  | 270.1570 |
|  | 778 |  |  |  |  |  | 233.6280 |

pp represents posterior probability in the BEB analysis

Sites identified by more than one ML method are underlined

^a^Twice the difference in log-likelihood values between models M1a and M2a

^b^Twice the difference in log-likelihood values between models M7 and M8

**p < 0.01
