# Supplementary material for: Adaptive Evolution of Toll-Like Receptors (TLRs) in the Family Suidae
Source: PLoS One. 2015 Apr 20;10(4):e0124069. doi: 10.1371/journal.pone.0124069 (PMC4404360; doi:10.1371/journal.pone.0124069)
Supplement: S3 Table — (DOCX) [file pone.0124069.s005.docx]

**Table S3.** Parameter estimates for PAML models used in detecting persistent positive selection in members of the family Suidae

| ***Gene*** | **Model** | **Parameters** | **Log likelihood** |
| --- | --- | --- | --- |
| *TLR1* | M1 | p_0_ = 0.7411, ω_0_ = 0.0000, p_1_ = 0.2589, ω_1_ = 1.0000 | -2640.9531 |
|  | M2 | p_0_ = 0.9843, ω_0_ = 0.2002, p_1_ = 0.0000, ω_1_ = 1.0000 | -2630.2586 |
|  |  | p_2_ = 0.0157, ω_2_ = 20.3745 |  |
|  | M7 | p = 0.0050, q = 0.0118 | -2641. 0997 |
|  | M8 | p_0_ = 0.9843, p = 24.8444, q = 99.0000, p_1_ = 0.0157, ω = 20.3864 | -2630.2590 |
| *TLR2* | M1 | p_0_ = 0.6628, ω_0_ = 0.0000, p_1_ = 0.3372, ω_1_ = 1.0000 | -2757.8967 |
|  | M2 | p_0_ = 0.9837, ω_0_ = 0.2757, p_1_ = 0.0000, ω_1_ = 1.0000 | -2753.9824 |
|  |  | p_2_ = 0.0163, ω_2_ = 11.1175 |  |
|  | M7 | p = 0.0050, q = 0.0115 | -2758. 0063 |
|  | M8 | p_0_ = 0.9837, p = 37.7613, q = 99.0000, p_1_ = 0.0163, ω = 11.1332 | -2753.9831 |
| *TLR3* | M1 | p_0_ = 0.7411, ω_0_ = 0.0000, p_1_ = 0.2589, ω_1_ = 1.0000 | -2640.9531 |
|  | M2 | p_0_ = 0.9843, ω_0_ = 0.2002, p_1_ = 0.0000, ω_1_ = 1.0000 | -2630.2586 |
|  |  | p_2_ = 0.0157, ω_2_ = 20.3745 |  |
|  | M7 | p = 0.0050, q = 0.0118 | -2641. 0997 |
|  | M8 | p_0_ = 0.9843, p = 24.8444, q = 99.0000, p_1_ = 0.0157, ω = 20.3864 | -2630.2590 |
| *TLR6* | M1 | p_0_ = 0.7018, ω_0_ = 0.0000, p_1_ = 0.2982, ω_1_ = 1.0000 | -2688.1462 |
|  | M2 | p_0_ = 0.9221, ω_0_ = 0.0000, p_1_ = 0.0000, ω_1_ = 1.0000 | -2680.0786 |
|  |  | p_2_ = 0.0780, ω_2_ = 6.7045 |  |
|  | M7 | p = 0.0050, q = 0.0117 | -2688. 1465 |
|  | M8 | p_0_ = 0.9221, p = 0.0050, q = 1.9056, p_1_ = 0.0780, ω = 6.7045 | -2680.0786 |
| *TLR7* | M1 | p_0_ = 0.9554, ω_0_ = 0.0692, p_1_ = 0.0446, ω_1_ = 1.0000 | -3979.8010 |
|  | M2 | p_0_ = 0.9554, ω_0_ = 0.0692, p_1_ = 0.0247, ω_1_ = 1.0000 | -3979.8010 |
|  |  | p_2_ = 0.0200, ω_2_ = 1.000 |  |
|  | M7 | p = 0.1901, q = 1.4764 | -3979.7424 |
|  | M8 | p_0_ = 1.0000, p = 0.1901, q = 1.4765, p_1_ = 0.0000, ω = 1.0000 | -3979.7424 |
| *TLR8* | M1 | p_0_ = 0.7371, ω_0_ = 0.0000, p_1_ = 0.2629, ω_1_ = 1.0000 | -3977.1489 |
|  | M2 | p_0_ = 0.8604, ω_0_ = 0.0000, p_1_ = 0.0000, ω_1_ = 1.0000 | -3974.8477 |
|  |  | p_2_ = 0.1397, ω_2_ = 2.3685 |  |
|  | M7 | p = 0.0050, q = 0.0119 | -3977.3868 |
|  | M8 | p_0_ = 0.8604, p = 0.0050, q = 2.8051, p_1_ = 0.1397, ω = 2.3686 | -3974.8477 |

p_0_ represents proportion of sites with ω < 1

p_1_ represents proportion of sites with ω = 1

p_2_ represents proportion of sites with ω > 1

p and q represent parameters of the beta distribution
